# A Hierarchical Mechanotransduction System: From Macro to Micro

**DOI:** 10.1002/advs.202302327

**Published:** 2023-12-25

**Authors:** Rong Cao, Huimin Tian, Yan Tian, Xianghui Fu

**Affiliations:** ^1^ Department of Endocrinology and Metabolism Center for Diabetes Metabolism Research State Key Laboratory of Biotherapy and Cancer Center West China Medical School West China Hospital Sichuan University and Collaborative Innovation Center Chengdu Sichuan 610041 China

**Keywords:** cancer, mechanical force, mechanobiology, mechanotherapy, mechanotransduction, metabolic disease, signaling transduction

## Abstract

Mechanotransduction is a strictly regulated process whereby mechanical stimuli, including mechanical forces and properties, are sensed and translated into biochemical signals. Increasing data demonstrate that mechanotransduction is crucial for regulating macroscopic and microscopic dynamics and functionalities. However, the actions and mechanisms of mechanotransduction across multiple hierarchies, from molecules, subcellular structures, cells, tissues/organs, to the whole‐body level, have not been yet comprehensively documented. Herein, the biological roles and operational mechanisms of mechanotransduction from macro to micro are revisited, with a focus on the orchestrations across diverse hierarchies. The implications, applications, and challenges of mechanotransduction in human diseases are also summarized and discussed. Together, this knowledge from a hierarchical perspective has the potential to refresh insights into mechanotransduction regulation and disease pathogenesis and therapy, and ultimately revolutionize the prevention, diagnosis, and treatment of human diseases.

## Graphical abstract

Mechanotransduction is a process of translating mechanical stimuli into biochemical signals and performs important regulatory functions at multiple levels, from molecules, subcellular structures, cells, tissues/organs, to whole‐body level. This review summarizes the physio‐pathological roles and mechanisms of mechanotransduction from macro to micro, emphasizes the significance of mechanotherapy, and discusses the interdisciplinary application, current challenges, and prospects.

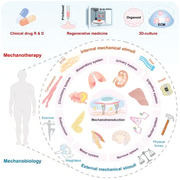

## 1 Introduction

Over decades, mechanical stimuli have been increasingly illuminated to play important physio‐pathological roles in many creatures’ life processes.^[^ ^1^, ^2^, ^3^ ^]^ Mechanotransduction is a process whereby creatures sense mechanical stimuli (inputs) and further transduce downstream biochemical responses (outputs).^[^ ^4^, ^5^ ^]^ Accumulating data suggest that mechanotransduction occurs across multiple biological scales. At the whole‐body level, many mechanical forces, such as gravity^[^ ^6^ ^]^ and exercise‐induced forces^[^ ^7^ ^]^ modulate systemic homeostasis. Subsequently, mechanotransduction has crucial roles in the development and function of tissues/organs.^[^ ^8^, ^9^, ^10^ ^]^ Meanwhile, diverse cellular processes, such as proliferation,^[^ ^11^, ^12^ ^]^ differentiation,^[^ ^13^, ^14^ ^]^ migration,^[^ ^15^, ^16^ ^]^ and apoptosis,^[^ ^17^, ^18^ ^]^ are regulated by various mechanical stimuli. At the molecular level, mechanical forces can alter the bond dynamics between molecules and the configurations of molecules,^[^ ^19^, ^20^ ^]^ which primes biochemical responses dictating numerous signaling pathways. Accordingly, aberrant mechanotransduction at different scales may promote the development of various diseases,^[^ ^21^ ^]^ and they may in return shift the mechanical context and mechanosensitive ability, facilitating a pathogenic vicious cycle that exacerbates disease progression.^[^ ^22^, ^23^ ^]^ Mechanotransduction thus provides new prospects for drug discovery and therapeutic manipulation, and is emerging as an attractive strategy for disease treatment.^[^ ^24^ ^]^ Despite the increasing awareness of mechanotransduction across different hierarchies, this burgeoning information has not been comprehensively reviewed, to our knowledge, which has paramount physio‐pathological and transformative significance and may ultimately be exploited to develop innovative therapeutic approaches.

Herein, we aim to systematically depict an integrated picture of the actions and mechanisms of mechanotransduction from macroscopic to microscopic scales, summarize their implications in human physiology and pathology, outline their applications in drug discovery and therapeutic manipulation, and discuss current challenges and future directions in fundamental and translational research.

## 2 Mechanical Stimuli in Multicellular Organisms

**Figure 1:**
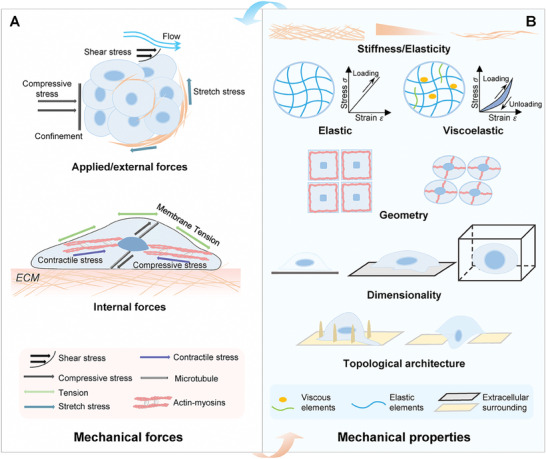
The classification of mechanical stimuli. Mechanical stimuli primarily comprise two types, namely mechanical forces (A) and mechanical properties (B). A) Mechanical forces can be yielded and exerted across many length scales in biology, for example, externally forces to tissues at the organ level, and internal forces generated by actomyosin motors within cells. B) Mechanical properties are behavioral or physical characteristics of materials or their surroundings, which are typically interwind with the forces. Indeed, when viewed as materials, living body components across different length scales exhibit either universal or unique mechanical properties. Collectively, different kinds of mechanical stimuli are not completely separative, but always interact with each other to yield an integrated physical information system.

**Figure 2:**
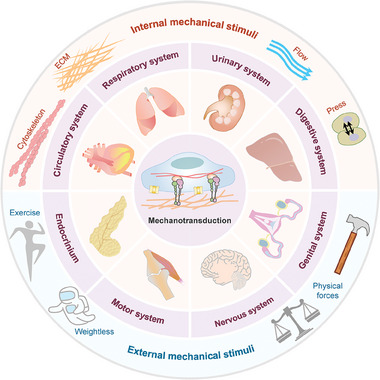
Mechanotransduction from macro to micro. From macro to micro, the human body is subjected to internal and external mechanical stimuli. External mechanical forces can result from movement, exercise, weightlessness, physical attacks, etc. Meanwhile, there are numerous internal mechanical forces in the human body, such as fluid shear force generated by heart pumping, interstitial fluid force, compressive stress originated from the extrusion between cells and extracellular matrix (ECM), etc. Mechanotransduction is essential to sense the various mechanical stimuli, thereby affecting multiple cellular, tissue/organ, and systemic biology. The online resource inside this figure was quoted or modified from Scienceslide2016 plug‐in.

In multicellular organisms, mechanical stimuli consist of diverse mechanical forces^[^ ^25^ ^]^ and properties^[^ ^26^, ^27^ ^]^ (**Figure** **1**), which are usually counter‐balanced and interact to offer complicated mechanical cues (see the next paragraph). Discerning the instructive information from these anfractuous cues is important for creatures to perform functions and maintain homeostasis, which are coordinately regulated by diverse molecules, cells, tissues, and organs (**Figure** **2**). Therefore, it is of significance to improve our understanding of mechanotransduction in a multiscale way.

### 2.1 The Intricate Relationships Among Mechanical Stimuli

There are intricate interactions among different mechanical stimuli, which contribute to the establishment of a mechanical informational system in multicellular creatures: 1) A battery of regulatory mechanisms can dynamically balance the internal and external forces;^[^ ^28^ ^]^ 2) Forces at the macro levels can channel along a tensegrity network to the micro levels;^[^ ^29^, ^30^ ^]^ 3) As a universal mechanical property, viscoelasticity actually delineates the effect of forces on mechanical properties;^[^ ^31^, ^32^ ^]^ 4) Viscoelasticity can also permeate the tensegrity network to influence force propagation,^[^ ^33^, ^34^ ^]^ possibly due to that viscoelasticity and tensegrity share common molecular mechanisms regarding energetic nonequilibrium‐to‐equilibrium transitions of bonds;^[^ ^35^, ^36^ ^]^ 5) The internal forces of cells and tissues are actually the key to the sensation of external mechanical properties.^[^ ^37^ ^]^

## 3 Mechanobiology at the Whole‐Body Level

Humans and animals living on earth are constantly subjected to various natural mechanical forces, including gravity and air pressure, as well as routine forces from exercise, which arouse a body‐level mechanobiology regulatory network. Here, gravity and exercise are used as a representative for natural and routine forces, respectively, to exemplify mechanobiology at the whole‐body level.

The paramount effects of gravity on systemic physiology are emphasized by spaceflight,^[^ ^6^, ^38^ ^]^ in which weightlessness or microgravity leads to changes in various mechanical stimuli, including increased intracranial pressure,^[^ ^39^, ^40^ ^]^ reduced blood pressure and cardiac contractility,^[^ ^41^, ^42^ ^]^ decreased mechanical load on skeleton,^[^ ^43^ ^]^ and other alternations in fluid distribution.^[^ ^6^ ^]^ As a result, the astronauts may experience multi‐systemic physio‐pathological changes,^[^ ^44^ ^]^ such as telomere elongation, neurovestibular deconditioning,^[^ ^45^ ^]^ spinal and skeletal dysfunction,^[^ ^46^ ^]^ reduced baroreceptor responses,^[^ ^47^ ^]^ altered immune capacity,^[^ ^44^ ^]^ cardiovascular dysfunction,^[^ ^48^ ^]^ and dysregulated metabolism.^[^ ^49^ ^]^ It is increasingly believed that multiscale mechanotransduction mechanisms contribute considerably to these microgravity‐related deviations,^[^ ^6^, ^44^, ^50^, ^51^ ^]^ albeit the underlying mechanisms remain largely unknown.

Exercise is known to provoke pleiotropic and myriad benefits, including skeletal muscle improvements,^[^ ^52^ ^]^ cardiovascular health,^[^ ^53^, ^54^ ^]^ tissue regeneration,^[^ ^55^ ^]^ bone homeostasis,^[^ ^56^ ^]^ and brain health,^[^ ^57^, ^58^ ^]^ which may be partially attributed to the effect of mechanical stimuli on tissues/organs.^[^ ^59^ ^]^ Far from gravity that generates relatively static stimuli, exercise gives birth to multi‐layer active mechanical stimuli, such as enhanced muscle contraction and fluid shear forces, which may induce organ‐specific mechanotransduction pathways. For example, muscle contraction can increase muscle protein synthesis to promote muscle hypertrophy by activating the mammalian target of rapamycin (mTOR) signaling pathway.^[^ ^7^, ^60^ ^]^ Moreover, mechanotransduction contributes to the secretion and multiplying of exerkines, a variety of bioactive substances released by multiple tissues including the muscle, liver, and adipose in response to exercise, which then improve systemic health via endocrine, paracrine, and autocrine actions.^[^ ^52^, ^58^, ^61^, ^62^, ^63^ ^]^

In response to weightlessness and exercise, diverse mechanical stimuli and signaling pathways are undoubtedly integrated to induce the synchronized changes in many tissues/organs. It is highly anticipated that our deepening understanding and the further development of approaches for dissecting systemic mechanobiology should help unravel tissue, cellular, and molecular networks essential for physiology and pathology.

## 4 Mechanobiology at the Embryonic Level

In some sense, embryo could be another excellent and simple model for deciphering the dynamics and integrations of systemic mechanobiology. Various mechanical stimuli, such as the tissue‐level contractility, viscoelasticity, and hydrodynamic forces, are crucial for embryogenesis, especially morphogenesis and differentiation.^[^ ^64^, ^65^ ^]^ On the one hand, tissue‐level actomyosin contractility plays a role in integrating different cells together, thus enabling the formation of the entire embryo.^[^ ^66^, ^67^ ^]^ On the other hand, this tissue‐level contractility drives many morphogenesis procedures, such as blastomere compaction,^[^ ^68^ ^]^ body‐axis formation,^[^ ^69^ ^]^ bending and invaginations,^[^ ^70^ ^]^ as well as the establishment of segregation or boundaries.^[^ ^71^ ^]^ For example, as the first important change in embryo morphogenesis, blastomere compaction at 8‐cell stage is partially regulated by the inter‐cellular tension generated by actomyosin contractility.^[^ ^68^ ^]^ Additionally, the polarized actomyosin contractility can advance the advent of dorsoventral and left‐right axes in vertebrate embryos.^[^ ^67^ ^]^ Other mechanical stimuli are also emerging in modulating embryogenesis. For instance, extracellular matrix (ECM) hyaluronate pressure is important for the formation of zebrafish semicircular canal,^[^ ^72^ ^]^ while forces from confinement and fluid flow are key for asymmetry establishment.^[^ ^73^, ^74^ ^]^ In fact, embryo morphogenesis is regulated by diverse mechanical stimuli as well as their interactions.^[^ ^72^ ^]^ The self‐organization of embryonic tissues can be directed by the topological defects and the gradients of compressive stresses.^[^ ^75^ ^]^ Likewise, external hydrodynamic forces and tissue elasticity concordantly drive the symmetry‐breaking events in embryo structures.^[^ ^76^ ^]^ However, more interactions between different mechanical stimuli and their contributions in embryogenesis await further investigation.

Mechanotransduction is central to sensing the mechanical stimuli and inducing biochemical changes occurring during embryogenesis.^[^ ^64^ ^]^ For instance, mechanical forces can activate the mechanosensitive transforming growth factor‐β (TGF‐β), thus triggering the expression of paired‐like transcription factor (Pitx2) on the left side of embryos to direct vertebrate gut rotation.^[^ ^77^ ^]^ Similarly, shear forces of leftward fluid flow induce the activation of mechanosensitive Ca^2+^ signaling in immotile cilia, thereby instructing the advent of left‐right asymmetry.^[^ ^73^, ^74^ ^]^ Notably, mechanical stimuli and mechanotransduction have important roles in cell differentiation/proliferation and inter‐cellular coordinative behaviors,^[^ ^78^ ^]^ and these processes could also tune different mechanical stimuli in embryos,^[^ ^79^ ^]^ indicating a long‐term feedback loop.^[^ ^80^ ^]^ Nonetheless, it is debated whether these mechanical stimuli and mechanotransduction pathways are pre‐dictated or occur stochastically. Additionally, it is of importance to decipher these mechanotransduction pathways and their connections at the tissue/organ level.

## 5 Mechanobiology at the Organ/Tissue Level

Mechanical forces and properties usually vary according to the functions, compositions, and structures of the organ/tissue. Cardiovascular tissues are subjected to blood shear forces and pressures, as well as cardiac contractility,^[^ ^81^ ^]^ respiratory organs particularly experience rhythmic forces including air shear and stretching forces, while gut–intestinal tract generates and experiences various forces during digesting food.^[^ ^82^, ^83^ ^]^ Increased ECM stiffness, cell tension, and cell density dictate a stiff tissue, and changes in the interstitial fluid and overall topology cause alterations in mechanical properties.^[^ ^84^, ^85^ ^]^

Mechanical stimuli can modulate the late development and regeneration of many tissues/organs. For instance, the proper formation of vascular tree requires modest hemodynamic forces,^[^ ^86^ ^]^ and thus low blood flows induce vascular regression.^[^ ^81^ ^]^ Similarly, intestinal loop morphogenesis is modulated by a set of mechanical properties containing tissue geometry, elasticity of the gut‐mesentery system, and forces from gut tube and dorsal mesentery.^[^ ^82^, ^87^ ^]^ Of note, the neural system plays a pivotal role in the sensation of mechanical stimuli,^[^ ^88^ ^]^ and mechanical stimuli can in return modulate neural system remodeling,^[^ ^89^ ^]^ indicating an interplay between mechanical stimuli and functional architecture of the neural system.

Expectedly, mechanical stimuli affect the function and physiology of mature organs/tissues. Elevated blood pressure leads to a long‐standing contraction of the arterial vessels with small resistance,^[^ ^81^ ^]^ while decreased blood flow induces a transient relaxation of the vessels,^[^ ^90^, ^91^ ^]^ thereby maintaining a relatively stable blood pressure. In the neural regulation processes, touch,^[^ ^92^ ^]^ poking, sound wave, digestion, and solid compression can trigger electrochemical responses in the neural circuit network and thus control peripheral functions.^[^ ^83^ ^]^ For example, mechanical stimuli can activate the enteric nervous system to produce electroneurographic signals, and relay them to the brain for feedback control of ingestion^[^ ^93^ ^]^ and digestion,^[^ ^94^ ^]^ ensuring proper function and homeostasis of the gastrointestinal tract.^[^ ^95^ ^]^

Mechanistically, the roles of mechanical stimuli on tissues/organs rely on mechanotransduction to regulate cell behavior.^[^ ^96^, ^97^ ^]^ Nevertheless, the mechanical stimuli usually differ depending on the locations and states of recipient cells,^[^ ^97^ ^]^ leading to cell‐specific mechanotransduction and biochemical responses. More importantly, the mechanobiological outcome at the organ/tissue level is an integration and coordination of mechanotransduction of individual cells.^[^ ^98^ ^]^ Therefore, the information on cellular mechanotransduction is a prerequisite for illustrating mechanotransduction at the organ/tissue level in detail.

## 6 Mechanotransduction at the Cellular Level

**Figure 3:**
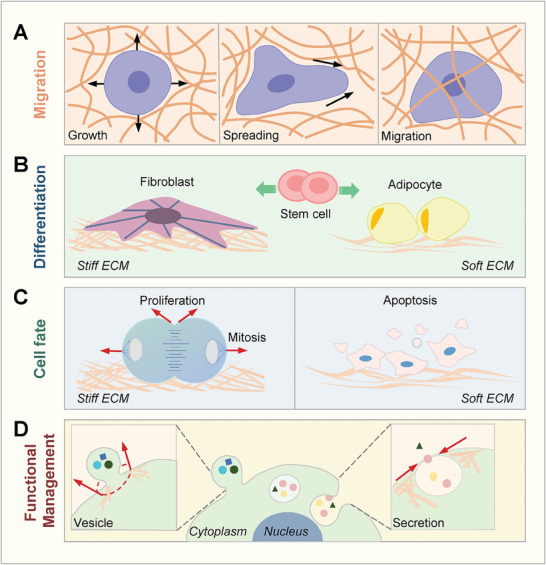
Mechanotransduction in cellular behaviors. A) Cell migration is attributed to various mechanical parameters, such as surrounding confinements, stiffness, and cell rear contraction. B) Stem cells exposed to differential mechanical stimuli are disparate in their differentiation prospects due to the different levels of activation of mechanotransduction pathways. C) Cell proliferation and division usually need favorable mechanical stimuli, while cells would undergo death upon losing effects from correct mechanical stimuli. D) Various cellular functions, such as secretory event and vesicle formation, are dependent of mechanical parameters (e.g., membrane tension and cytoskeletal forces) and the activation of numerous mechanotransduction pathways.

Cellular mechanotransduction is a process by which cells sense and convert mechanical stimuli into biological responses, modulating cellular behaviors in tissue development and functions^[^ ^99^ ^]^ (**Figure** **3**).

### 6.1 Mechanotransduction in Cell Migration

Cell migration, including single‐cell and collective migration, has been intensively studied in cellular mechanotransduction, due to its intimate association with many mechanical parameters.^[^ ^15^ ^]^ To migrate properly, cells need to connect these mechanical parameters to diverse subcellular activities (Section 6.1.1), thereby adopting differential migration manners, e.g., mesenchymal, lobopodial, amoeboid, and collective migration.^[^ ^100^ ^]^

#### 6.1.1 Cell Migration is Regulated By Various Mechanical Parameters and Subcellular Activities

Cell migration is not only related to the regulation of a bevy of mechanical parameters, including intracellular force, ECM stiffness or viscoelasticity, dimensionality, physical confinement, and topologic architectures, but also to a battery of subcellular mechanical activities, including the remodeling of cytoskeletal architecture, fluctuations of cytoskeletal forces, and transformations of integrin‐adhesions.^[^ ^101^, ^102^, ^103^ ^]^ Briefly, lamellipodial or filopodial actin polymerization couples with NAs to drive the protrusion of cell leading edge, while contractile force of actomyosin associated with FAs in the lamellum (behind the leading area) pulls the cell towards migrating direction, accompanied by disconnection of fibril (loosened) adhesions from underlying ECM.^[^ ^104^ ^]^

#### 6.1.2 Single Cell Migration

Mechanotransduction mediated by integrin‐adhesions, cytoskeleton, and nucleus, as well as their interplays, is crucial for single‐cell migration. Cell cytoskeleton can generate contractile forces that then transmit along integrin‐adhesions to apply on the ECM, providing anchor sites for the cell to further migrate.^[^ ^104^, ^105^ ^]^ In this process, integrin‐adhesions can sense the forces transmitting along them and finally induce changes in adhesion dynamics^[^ ^106^, ^107^, ^108^ ^]^ and activation of various signaling pathways, such as focal adhesion kinase (FAK)^[^ ^109^ ^]^ and Rho (GTPase)/Rho‐associated protein kinase (ROCK) pathways.^[^ ^110^ ^]^ However, different cells usually rely on different cytoskeletal forces and dynamics to migrate, and the prime type of cytoskeletal mechanotransduction in migration may be therefore distinct in different cells. When cell migration is mainly driven by actin cytoskeleton, forces from actin polymerization or actomyosin contraction can activate the actin‐related protein2/3 (**Arp2/3**) **complex** to maintain long‐term migration.^[^ ^111^ ^]^ For cell migration dependently of microtubule, microtubule acetylation and polymerization can scale with ECM stiffness and cell tension, and further regulate actomyosin contractility in migration.^[^ ^101^, ^112^ ^]^ For cell migration through a confined environment, the nucleus could be deformed by increased cytoskeletal forces,^[^ ^113^, ^114^ ^]^ which regulates gene expression regarding adhesion and cytoskeleton and thus adjusts the cell mechanics and migration strategy to adapt to the confinement.^[^ ^115^, ^116^, ^117^ ^]^

Single cell migration usually has specific paths depending on the landscape of extracellular signals and mechanisms, such as chemotaxis, haptotaxis, durotaxis, and topotaxis,^[^ ^118^ ^]^ by which require the generation, the sensation, the transmission, and the execution of relevant signals.^[^ ^118^ ^]^ In durotaxis^[^ ^119^, ^120^ ^]^ and topotaxis,^[^ ^121^ ^]^ mechanotransduction processes generated at integrin‐adhesion, cytoskeleton, and nucleus, as well as their integration, play indispensable roles in sensing, transmitting, and transducing the matrix stiffness and topological cues,^[^ ^106^, ^118^ ^]^ albeit the underlying mechanisms are still unclear. It is also worthy of investigating how cells respond to multiple mechanical stimuli at the same time through mechanotransduction.

#### 6.1.3 Collective Migration

In addition to the aforementioned mechanotransduction in single‐cell migration, collective migration is in particular related to the mechanical stimuli generated from cell–cell and cell–matrix adhesions. During collective migration, cell–cell adhesions bear the highest stresses to ensure the integration of the collective cells^[^ ^9^ ^]^ and the generation of tissue‐level forces.^[^ ^8^ ^]^ Moreover, cell–cell adhesions can modulate cell contractility and polarization dependently on the differential traction forces between the leader and follower cells,^[^ ^122^ ^]^ thereby enabling collective migration with minimal intercellular shear stress.^[^ ^123^ ^]^ On the other hand, cell–matrix adhesions may affect intercellular communications^[^ ^35^, ^98^ ^]^ and regulate collective migration by controlling ECM remodeling and mechanics.^[^ ^120^, ^124^ ^]^ Interestingly, the intercellular propagation of intracellular signaling molecules is also sufficient to modulate collective migration by altering cell mechanics.^[^ ^125^ ^]^ Hence, whether there is a bidirectional mechanochemical coupling in collective cell migration may be a focus of future research.

### 6.2 Mechanotransduction in Cell Differentiation

Mechanotransduction plays an important role in cell differentiation by regulating morphology specification and fate determination.^[^ ^78^ ^]^ Generally, high ECM stiffness can cause a spreading and flat cell phenotype by increasing the density and size of integrin‐adhesions,^[^ ^126^ ^]^ actomyosin contractility,^[^ ^14^, ^127^ ^]^ and nuclear stiffness.^[^ ^128^ ^]^ By contrast, soft substrate could guide stem cells to display a thick and round morphology.^[^ ^129^ ^]^ Similar to morphological responses, stem cells on a rigid substrate usually differentiate to myoblasts, osteoblasts, and fibroblasts, while those on a soft substrate preferentially differentiate to adipocytes and neurons^[^ ^130^, ^131^ ^]^ (Figure 3). Mechanistically, high ECM stiffness can activate integrin‐adhesion/extracellular signal‐regulated kinase (ERK) signaling pathways to modulate stem cell fate,^[^ ^132^ ^]^ genomic architecture, and transcriptional profiling.^[^ ^133^ ^]^ Enhanced stiffness could also promote nuclear accumulation of yes‐associated protein/transcriptional co‐activator with PDZ‐binding motif (YAP/TAZ)^[^ ^99^ ^]^ and myocardin‐related transcription factor/serum response factor (MRTF/SRF)^[^ ^134^ ^]^ via the induction of integrin‐adhesion maturation, actomyosin contractility, cytoskeleton assembly, and nuclear pore complex (NPC) opening, collectively fostering transcriptional programs promoting differentiation.^[^ ^135^, ^136^ ^]^

Other mechanical stimuli, such as membrane tension,^[^ ^137^ ^]^ mechanical compression,^[^ ^138^ ^]^ fluid flow forces,^[^ ^139^ ^]^ and asymmetric cytoskeletal force,^[^ ^140^, ^141^ ^]^ also influence cell morphology and differentiation.^[^ ^99^ ^]^ In general, the prime mechanotransduction pathways sensing these mechanical stimuli are not always identical to those sensing ECM stiffness. For example, membrane tension is primarily sensed by caveolae^[^ ^142^ ^]^ and ion channels,^[^ ^137^ ^]^ and thus modulates several differentiation‐related signaling pathways, such as caveolin pathways and Ca^2+^/calmodulin‐dependent protein kinases (CaMKs)/ c‐Jun N‐terminal kinase (JNK). Notably, mechanotransduction pathways may vary according to the types and states of stem cells. For example, in response to high contractility, human mesenchymal stem cells adopt the ERK and WNT pathways to initiate osteogenic differentiation,^[^ ^143^ ^]^ while mouse pluripotent stem cells rely on TGF‐β activation to promote endodermal differentiation.^[^ ^144^ ^]^

### 6.3 Mechanotransduction in Cell Proliferation

Mechanotransduction functions in cell proliferation by regulating proliferation‐related transcription cofactors, cell cycle entry, and cell division (Figure 3).^[^ ^145^ ^]^ As important transcription cofactors, nuclear localization of YAP/TAZ plays a crucial role in cell proliferation.^[^ ^136^, ^146^ ^]^ Mechanical signals could activate the integrin/FAK pathway to enhance contractility and dynamics of actin‐cytoskeleton, thereby driving YAP/TAZ nuclear localization and cell proliferation.^[^ ^147^, ^148^ ^]^ By contrast, in cells experiencing low mechanical stresses, the YAP/TAZ activity is reduced by some actin‐binding proteins, such as filamin, cofilin, actin‐capping protein Z (CapZ) and myosin II, thereby inhibiting cell proliferation.^[^ ^145^ ^]^ A recent study revealed that YAP/TAZ only instructs the proliferation of cells exposing to mechanical stresses,^[^ ^149^ ^]^ further deepening the significance of mechanotransduction in controlling YAP/TAZ‐related cell proliferation.

Cell cycle entry and division are also associated with mechanotransduction. It was shown that increased ECM stiffness activated the FAK/Cas/ Ras‐related C3 botulinum toxin substrate 1 (Rac) pathway and promoted cyclin D1 expression and therefore S‐phase entry.^[^ ^150^ ^]^ Likewise, enhanced force could upregulate β‐catenin activation and cyclin D1 expression via cadherin‐adhesion.^[^ ^151^ ^]^ During cell division, Piezo 1 can be activated by the enhanced membrane tension, and then facilitates the ERK1/2‐dependent transcription of cyclin B^[^ ^152^ ^]^ and the activation of protein kinase B (AKT)/CyclinD1 pathway.^[^ ^153^ ^]^ Another crucial movement in cell division—chromosome separation, requires a faithful mechanical transport elicited by microtubule‐based mitotic spindles.^[^ ^154^ ^]^ Specifically, tensile and compressive forces applied on microtubules could respectively enhance and subside the polymerization of microtubules and the attachment of kinetochore to microtubules.^[^ ^155^ ^]^ These outcomes, as well as architectural changes of mitotic spindles caused by rotational and sliding forces, are key to the reorganization of chromosomes in mitosis events.^[^ ^155^ ^]^ In these processes, forces also affect epigenetic modifications and chromosomal states, further altering long‐term genomic status.^[^ ^156^, ^157^ ^]^ Additionally, during cytokinesis, caveolae can promote endosomal sorting complex required for transport‐III (ESCRT‐III) assembly and cytokinetic abscission by buffering membrane tension and limiting the interface contractility in dividing cells.^[^ ^158^ ^]^ It is of great interest to investigate the possibility of inheritable mechanotransduction. For example, whether mechanotransduction‐induced genomic alternations in parent cells could shape daughter cell mechanosensitivity or not.

### 6.4 Mechanotransduction in Cell Death

Mechanotransduction can control cell death in many ways (Figure 3).^[^ ^18^ ^]^ It has been well‐recognized for a long time that cells on substrates with extremely low stiffness undergo apoptosis called anoikis.^[^ ^159^ ^]^ Mechanistically, low stiffness can depress some pro‐survival signaling pathways, such as ERK, JNK, p21‐activated protein kinase 2 (PAK2),^[^ ^160^ ^]^ and AKT, to activate caspases and DNA fragmentation, ultimately inducing anoikis.^[^ ^161^ ^]^ Cell apoptosis is also induced by the compressive stress in epithelial monolayer through the inactivation of YAP and the activation of caspase‐3.^[^ ^18^ ^]^ In addition to apoptosis, ferroptosis is emerging to relate with mechanotransduction through a variety of pathways,^[^ ^162^, ^163^, ^164^ ^]^ despite of the elusive underlying molecular pathways.

### 6.5 Mechanotransduction in Cell Metabolism

Mechanotransduction is emerging to regulate a diversity of cell metabolic proceedings, including glucose uptake,^[^ ^165^ ^]^ glycolysis,^[^ ^166^ ^]^ the transition from glycolysis to oxidative phosphorylation system (OXPHOS),^[^ ^137^ ^]^ metabolic redox homeostasis,^[^ ^167^ ^]^ lipid metabolism^[^ ^168^ ^]^ and others.^[^ ^169^ ^]^ Typical mechanosensitive subcellular structures and molecules, such as cadherin‐adhesion,^[^ ^170^ ^]^ cytoskeleton^[^ ^171^ ^]^ and mechanosensitive channel,^[^ ^137^, ^172^ ^]^ are also involved in these metabolic responses.^[^ ^169^ ^]^ YAP/TAZ serve as an essential node in modifying the metabolic availability of cells under mechanical stimuli.^[^ ^173^ ^]^ Intriguingly, mechanical stimuli can also change the morphologies and structures of the golgi apparatus and mitochondria by regulating cytoskeletal dynamics and forces, offering attractive links to cellular metabolic states.^[^ ^167^, ^174^, ^175^ ^]^ In addition, mechanotransduction plays a role in macrophage polarization,^[^ ^176^ ^]^ flow‐mediated dilation of endothelial cells,^[^ ^177^ ^]^ mechanical pain^[^ ^178^ ^]^ and itch sensation^[^ ^179^ ^]^ of neurons, insulin secretion of β cells,^[^ ^180^, ^181^ ^]^ vesicle formation,^[^ ^182^, ^183^ ^]^ etc., greatly expanding the scope of cellular mechanotransduction.

**Figure 4:**
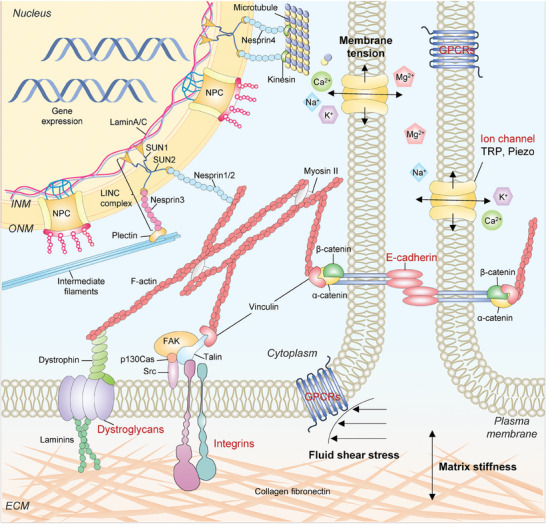
Mechanotransduction at the subcellular level. Forces are transmitted from ECM to nucleus and then converted to biochemical signals to regulate cellular behaviors and functions. Typically, the mechanotransduction pathway is activated by three steps. First, mechanosensors (highlighted in RED) sense mechanical stimuli and transmit them into the cell. Subsequently, cytoskeletons, including F‐actin, intermediate filaments, and microtubules, are connected to the internal nuclear envelope through the LINC complex for force delivery to the nucleus. Finally, nuclear mechanosensors transmit mechanical stimuli into nuclear signals and regulate gene expression.

**Figure 5:**
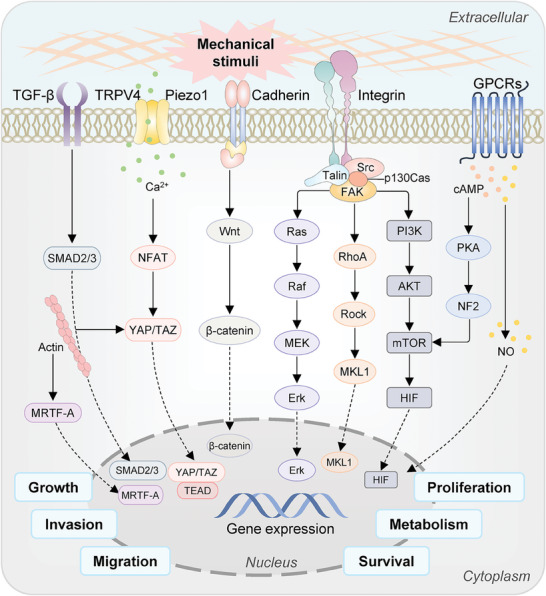
Key signaling pathways in mechanotransduction. Mechanosensors on the cell membrane could sense mechanical stimuli and then convert them into biochemical signals, subsequently activating downstream signaling pathways, such as the Ras, RhoA/Rock, YAP/TAZ, PI3K/AKT, and Wnt/β‐catenin, etc. Only a few pathways are illustrated here in different colors. Notably, the contributions of mechanotransduction‐mediated biochemical responses are orchestrated and maintained by diverse signaling interactions. They will selectively activate transcription factors, modulate gene expression, and ultimately regulate cellular behaviors, including growth, proliferation, survival, invasion, migration, and metabolism.

Overall, in cellular mechanotransduction, various subcellular and molecular mechanotransduction procedures are intricately coupled to regulate the dynamics and architectures of diverse subcellular structures, the shuttling of transcriptional factors, the transduction of signaling molecules, and gene activities (**Figures** **4** and **5**). Moreover, cellular mechanotransduction usually spans over different timescales and throughout the entire cell, which are critical for modulating tissue dynamics, yet how cellular mechanotransduction works in the tissue context remains poorly understood. Additionally, it holds great significance to enquire how cells choose distinct mechanotransduction strategies to acclimatize themselves with different mechanical contexts, and whether these choices are encoded by the genes or shaped by the co‐evolving relationship between the cell biology and mechanics.

## 7 Mechanotransduction at the Subcellular and Molecular Levels

**Figure 6:**
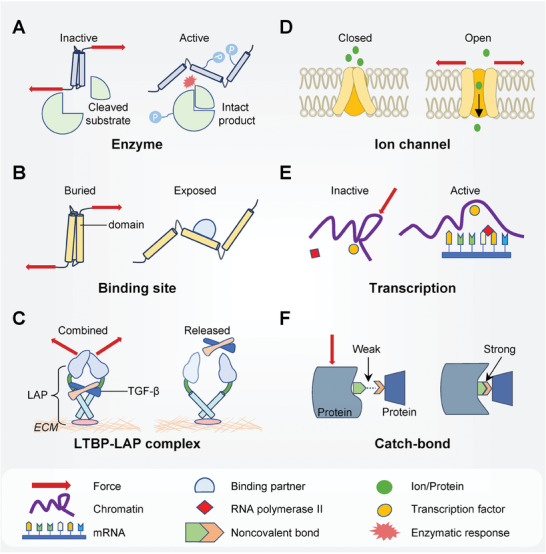
Diagram of examples on molecular mechanotransduction. Molecular mechanotransduction could lead to the force‐induced conformational changes in a single protein and the ensuing biological response. A) Force‐induced conformational changes of the enzymes (e.g., FAK) may upregulate the catalytic activity or facilitate the phosphorylation of the molecules. B) Force could unfold inactive proteins (e.g., talin) to expose previously concealed binding sites for the partner proteins. C) Owing to force‐induced conformational changes, hosting proteins (e.g., LAP) can dissociate from their previously fastened proteins (e.g., TGF‐β). D) The opening of mechanosensitive channels across the cellular or nuclear membranes may be responsive to membrane tension, allowing for the shuttling of ions or proteins. E) Forces can decondense chromatin, aiding the proximity of transcription factors and RNA polymerase II to the gene locus, thereby altering the transcriptional state. F) Schematic of the allosteric model for catch‐bond. The applied force can induce conformational changes that underpin pre‐existing noncovalent bonds between the protein and its binding partner.

Owing to ‘force‐induced conformational change in single protein’ and ‘catch‐bond’ mechanisms (**Figure** **6** and Section 7.1), molecules can sense forces and produce molecular responses wired to mechanotransduction at higher scales.^[^ ^20^, ^184^ ^]^ Notably, these molecular mechanisms interact with each other, which is also regulated by force characteristics and molecular sensitivities.^[^ ^185^ ^]^ As such, force‐sensitive molecules can serve as band‐pass filters to exclude mechanical noise and discern a range of physiologically relevant forces, providing mechanistic underpinnings for subcellular mechanotransduction.

### 7.1 Molecular Mechanisms for Force Sensation

#### Force‐Induced Conformational Changes in a Single Protein

Certain proteins, due to their specialized mechanical architectures/stability, free‐energy landscapes, and spatial positions, have inherent abilities to measure forces through conformational changes.^[^ ^19^, ^20^, ^184^, ^186^, ^187^ ^]^ Molecules with appropriate mechanical stabilities could avoid being directly ruptured by forces.^[^ ^186^ ^]^ Meanwhile, according to free‐energy landscapes, certain forces can facilitate the activation of molecular conformations by lowering the energy barriers between inactive and active conformations, and further stabilizing the molecules in the active conformations.^[^ ^19^, ^188^, ^189^ ^]^ Additionally, molecules have preferences over their structures and spatial positions, sensitizing their likelihood of conformational changes to the magnitude, duration, direction, and rates of forces.^[^ ^19^, ^185^, ^188^, ^189^ ^]^ Usually, force‐induced conformational changes could bring about a diversity of biochemical responses in molecules, ranging from altered enzymatic activity,^[^ ^12^, ^190^, ^191^, ^192^ ^]^ exposure of cryptic binding sites,^[^ ^193^, ^194^, ^195^, ^196^, ^197^ ^]^ release of their binding proteins,^[^ ^198^, ^199^, ^200^, ^201^, ^202^ ^]^ phosphorylation^[^ ^203^ ^]^ or dephosphorylation,^[^ ^129^, ^204^, ^205^ ^]^ channel opening,^[^ ^206^, ^207^, ^208^, ^209^, ^210^ ^]^ to alterations in gene expression^[^ ^211^, ^212^ ^]^ (Figure 6).

#### Catch‐Bonds Between Proteins

Non‐covalent bonds between proteins usually have certain lifetimes ranging from milliseconds to days,^[^ ^188^ ^]^ and the majority of those would shorten lifetimes in response to force application and thus lead to the dissociation of protein‐complexes, whereby namely ‘slip‐bonds’.^[^ ^185^, ^213^ ^]^ Yet, some inter‐molecular bonds prolong their lifetimes in response to forces of appropriate magnitudes and durations, thereby strengthening protein‐protein interactions, whereby namely ‘catch‐bond’.^[^ ^213^, ^214^ ^]^ Catch‐bonds widely exist in cytoskeleton, adhesions, and transmembrane interactive structures.^[^ ^215^ ^]^ Some models have been purposed to elucidate the mechanism for catch‐bonds.^[^ ^213^ ^]^ The ‘allosteric regulation model’ is among the most approved, in which mechanical forces enhance protein‐protein binding states via the induction of protein conformational changes (Figure 6). The quantitative model of ‘two‐state catch‐bond’ delineates that moderate forces could eliminate the energy barrier between the weaker and stronger binding states and further stabilize stronger binding states.^[^ ^213^ ^]^ Intriguingly, catch‐bonds might have different mechanisms and energetic features, possibly due to differential molecular architectures and force‐sensitivities.

### 7.2 Cell‐Matrix Adhesion Mechanotransduction

Cell–matrix adhesion (integrin‐adhesion) mechanotransduction is nearly the most well‐studied subcellular mechanotransduction, and regulates various cellular and tissue behaviors as noted before.^[^ ^108^, ^185^ ^]^ Integrin‐adhesion has a 3D multilaminar architecture and dynamic compositions, which is key to complete mechanotransduction (Section 7.2.1), by which the molecular clutch model and other mechanisms are the underlying mechanisms.

#### 7.2.1 3D multilaminar Architecture of Integrin‐Adhesion

Integrin‐adhesion establishes a connection between ECM and actin cytoskeleton within cells, and mainly encompasses two subclasses: nascent NA and focal FA.^[^ ^185^ ^]^ Since NA is an immature FA, herein we will mainly focus on the role of FA.^[^ ^216^, ^217^ ^]^ On the plasma membrane, FA proteins have a 3D multi‐layer structure and are stratified into three functional layers. Near plasma membrane is the integrin signaling layer, where talin head domain and signaling‐related proteins, such as FAK, non‐receptor tyrosine kinase (Src), and Cas,^[^ ^218^, ^219^ ^]^ are co‐localized with integrin cytoplasmic tails.^[^ ^190^, ^216^ ^]^ Next is the force transduction layer, in which talin and vinculin reside to connect integrin to actin and thus transmit forces.^[^ ^105^ ^]^ Far from plasma membrane is the actin regulatory layer, in which actin‐binding proteins localize to regulate F‐actin dynamics.^[^ ^220^, ^221^ ^]^ Additionally, FA is compositional dynamic,^[^ ^222^, ^223^ ^]^ as most FA components constantly shuttle between their cytoplasmic pool and FA sites.^[^ ^185^ ^]^

#### 7.2.2 The molecular clutch model

Once the molecular clutch in focal adhesion (FA) is established (see the next paragraph), it could underpin the bi‐directional force transmission and initiate various bond dynamics. First, the force along clutch linkage can shift the conformation of integrin to a fully and long‐term active one, wherein namely mechanosensitive activation of integrin.^[^ ^224^, ^225^ ^]^ Second, during this process, the bond between integrin and arginine‐glycine‐aspartate (RGD) domain of fibronectin (FN) also transforms into a long‐term bond (catch‐bond), as observed in α_5_β_1_ integrin‐FN and α_v_β_3_ integrin‐FN.^[^ ^226^, ^227^ ^]^ These changes further lead to increased integrin affinity for ligands, long‐activation of integrin,^[^ ^228^ ^]^ and clustering of integrin.^[^ ^224^ ^]^ Nevertheless, it was noticed even when integrin bonds behave as slip‐bonds, the clutch still works properly,^[^ ^229^ ^]^ on the condition that new linkages between integrin and ECM ligands can rapidly arise to compensate slip‐bonds.^[^ ^109^ ^]^ Finally, forces also induce a conformational change of talin, facilitating the recruitment of vinculin^[^ ^230^ ^]^ and spurring various downstream responses, such as vinculin clustering and activation of signaling pathways.^[^ ^193^, ^194^ ^]^

#### The Establishment of Molecular Clutch in FA

FA can establish a mechanical “clutch” linkage in a subtle way. In lamellipodia, the branched F‐actin with a fast but short‐living retrograde movement driven by the Arp2/3 complex, interacts with the front tip of FA,^[^ ^231^ ^]^ while in lamellum, the stress fibers with a slow but long‐living retrograde movement driven by Myosin IIs, interacts with the back tip of FA.^[^ ^232^ ^]^ Meanwhile, the rapid exchange of talin between FA and cytoplasmic pool causes talin's transient residing between the moving actin flow and rare full‐active integrin.^[^ ^224^ ^]^ In this case, if the ECM ligand, e.g.,FN, concurrently binds to integrin,^[^ ^224^ ^]^ a long‐term actin‐talin‐integrin‐ligand clutch linkage (molecular clutch linkage) would emerge.^[^ ^107^ ^]^

In response to mechanical stimuli, e.g., ECM stiffness, the clutch model could regulate the force transmission and transduction at the integrin‐adhesion.^[^ ^105^, ^109^, ^233^ ^]^ For instance, if ECM is not excessively soft and ECM ligands are enough, a constant intracellular force could transmit to and deform ECM, resulting in a fast and progressive force loading along the clutch linkage.^[^ ^107^ ^]^ If ECM stiffness is comparable to intracellular force, force loading would become fast enough to allow talin to unfold before it unbinds,^[^ ^107^ ^]^ and trigger the catch‐bond behavior of integrin‐FN.^[^ ^227^ ^]^ Nonetheless, if ECM stiffness surpasses a high threshold (≈1300 Kpa), the loading becomes too strong to break the clutch linkage before new molecules binding, impeding force transmission and generating the outcomes similar to those on an excessively soft ECM.^[^ ^233^ ^]^ Provided that ECM is excessively soft, or available ECM ligands are little, or intracellular force is short‐lived, force loading will be very slow and small. Subsequently, talin unbinding precedes before active integrin stabilization occurs, hindering talin unfolding and catch‐bond behaviors.^[^ ^105^ ^]^ As a result, clutch molecules could disengage and fail to conduct function, causing a faster actin flow, reduced traction forces, repressed adhesion maturation, and inactivation of other biochemical signals.^[^ ^107^, ^233^ ^]^

#### 7.2.3 Other Molecular Mechanisms

Integrin‐adhesion mechanotransduction also necessitates the involvement of other molecular mechanisms. In detail, forces along the clutch linkage can partially activate FAK by changing its conformation.^[^ ^190^ ^]^ Partially activated FAK further recruits non‐Src for its full‐activation and forms a FAK‐Src complex to phosphorylate Cas and paxillin.^[^ ^190^ ^]^ Thereafter, Cas phosphorylation primes the activation of pathways involving Rac^[^ ^12^ ^]^ and Rap1,^[^ ^234^ ^]^ while paxillin phosphorylation promotes Crk binding^[^ ^235^ ^]^ and regulates adhesion dynamics.^[^ ^236^ ^]^ Particularly, the persistent force along paxillin‐FAK linkage may also facilitate the docking of growth factor receptor bound protein 2 (Grb2) to FAK,^[^ ^237^ ^]^ thereby coupling the FAK with Ras/mitogen‐activated protein kinases (MAPK) pathways.^[^ ^124^ ^]^ Notably, FAK also controls the activation of Rho/ROCK,^[^ ^238^ ^]^ actin remodeling,^[^ ^134^ ^]^ FA dynamics,^[^ ^106^ ^]^ and nucleocytoplasmic shuttling of YAP/TAZ^[^ ^147^ ^]^ and MRTF/SRF.^[^ ^134^ ^]^ Cas (especially p130Cas) also changes its configuration under forces, priming the activation of downstream signaling pathways, such as Rac and GTPase (e.g., Rap1).^[^ ^239^ ^]^ Besides, the kinetics of zyxin,^[^ ^220^ ^]^ α‐actinin,^[^ ^240^ ^]^ kindlin,^[^ ^224^ ^]^ and parvin^[^ ^218^ ^]^ are responsive to mechanical stimuli, regulating integrin‐adhesion mechanotransduction.^[^ ^107^ ^]^

In conclusion, owing to the clutch model and other molecular mechanisms, cell–matrix adhesions can sense extracellular and intracellular mechanical stimuli, and therefore adjust the kinetics of adhesions, cytoskeletal organization, and contractility, ECM composition and architecture, intracellular signaling pathways, and shuttling of transcription cofactors.^[^ ^16^, ^104^, ^108^ ^]^ These biochemical outcomes further control cellular and tissue mechanics and behaviors,^[^ ^16^ ^]^ giving rise to mechanotransduction stimulus‐response loops in cellular and tissue behaviors.^[^ ^241^ ^]^

### 7.3 Cell–Cell Adhesion Mechanotransduction

Cell–cell adhesion mechanotransduction enables multiple layers of cell–cell communications^[^ ^242^ ^]^ by regulating adhesion kinetics^[^ ^243^ ^]^ and various signaling pathways.^[^ ^151^ ^]^ Cadherin‐adhesion (CA) or adheren‐junction (AJ)^[^ ^243^, ^244^ ^]^ is among the most common cell–cell adhesions, consisting of cadherins and anchoring proteins including α‐catenin, β‐catenin, and vinculin. In CA, two ectodomains of cadherins connect together in *cis* and *trans* manners, while the anchoring proteins connect cadherin with intracellular actin cytoskeleton,^[^ ^245^ ^]^ providing structural foundation for mechanotransduction.

Mechanistically, catch‐bonds and molecular force‐sensitive conformational changes might concertedly induce changes of CA dynamics.^[^ ^243^ ^]^ Rakshit et al. uncovered that two *trans* cadherins of X‐dimer form a catch‐bond under moderate forces, whereas the bond becomes a slip‐bond under force over 30 pico‐Newton (pN), suggesting a biphasic catch‐slip bond behavior of X‐dimer cadherins.^[^ ^246^ ^]^ Similarly, α‐catenin–actin bond shows a “catch‐bond” behavior, thus resisting the force‐induced breakdown of CA‐F‐actin linkage.^[^ ^243^ ^]^ Meanwhile, force‐induced conformational change of α‐catenin facilitates the recruitment of vinculin to CA.^[^ ^195^ ^]^ Vinculin‐actin bond is a directionally asymmetric catch‐bond, because its lifetime prolongs in response to intermediate range (≈7–10 pN) forces and is sensitive to the force direction, which is key to polarizing F‐actin.^[^ ^247^ ^]^ The clutch model is speculated to coordinate the above force‐sensitive bond dynamics and molecular unfolding in CA mechanotransduction.^[^ ^107^, ^248^ ^]^

CA mechanotransduction also impacts the activation of signaling pathways.^[^ ^242^ ^]^ For example, CA of high localized tension can release β‐catenin^[^ ^133^ ^]^ via slip‐bond,^[^ ^249^ ^]^ while YAP can be liberated upon CA being dismantled.^[^ ^250^ ^]^ In response to high local stress, CAs may activate the AMP‐activated protein kinase (AMPK) pathway by regulating the liver kinase B1 (LKB1), linking metabolism to local mechanical status.^[^ ^170^ ^]^ In addition, CA mechanotransduction is able to control the recruitment of diverse actin regulators, e.g., Arp2/3 and formins, as well as the activation of GTPases and Src‐family pathways, thereby regulating cytoskeletal contractility and organization.^[^ ^8^ ^]^ Due to these mechanisms and outcomes, CA could modulate the mechanics and behaviors of neighbor cells and finally tune the intercellular communications in tissue dynamics and mechanotransduction.

### 7.4 Cytoskeletal mechanotransduction

Cytoskeleton is mainly subdivided into the microfilament (F‐actin or actin cytoskeleton), microtubule, and intermediate filament. Mechanotransduction can be generated at diverse kinds of cytoskeleton and regulate the adhesion‐cytoskeleton architecture, the activation of signaling pathways, and the transportation of transcription factors.^[^ ^111^ ^]^

#### 7.4.1 F‐actin

In F‐actin mechanotransduction, forces of individual actomyosin filaments integrate into a network‐level tension throughout the cell,^[^ ^251^ ^]^ giving rise to multiple layers of regulatory mechanisms and outcomes,^[^ ^252^ ^]^ accompanied by the involvement of strain‐stiffening^[^ ^253^ ^]^ or strain‐softening.^[^ ^254^ ^]^

First, individual actin filaments always bear forces deriving from plasma membrane compression and geometrical entanglement of the network.^[^ ^255^ ^]^ These forces may alter actin affinity for its binding to regulatory proteins^[^ ^256^ ^]^ by governing actin configuration,^[^ ^255^ ^]^ and thus modulate F‐actin architecture.^[^ ^257^ ^]^ Second, several actin binding proteins including filamin A,^[^ ^258^ ^]^ myosin II,^[^ ^259^ ^]^ α‐actinin 4,^[^ ^260^ ^]^ gelsolin,^[^ ^261^ ^]^ filamin B,^[^ ^260^ ^]^ formins,^[^ ^262^ ^]^ and WAVE regulatory complex (WRC),^[^ ^111^ ^]^ are per se targets of mechanical forces. Upon sensing forces, filamin A releases the prebound FilGAP to activate Rac.^[^ ^263^ ^]^ Furthermore, other actin‐binding proteins, such as myosin II, α‐actinin 4, and filamin B, may accumulate under enhanced cortical tension to upregulate cytoskeletal resistance to deformation.^[^ ^260^ ^]^ Conversely, WRC in lamellipodium decays in response to increased local stress, thereby activating the Arp2/3 complex and altering branched actin networks. Moreover, forces may modify formin (mDia1) activity to influence the elongation rate of F‐actin.^[^ ^262^ ^]^ Meanwhile, in the absence or presence of profilin, small forces respectively hinder or enhance actin polymerization governed by another formin, Bni1p.^[^ ^264^ ^]^ Forces also affect the binding affinity of gelsolin for Ca^2+^ ions, facilitating the recruitment of Ca^2+^ to F‐actin regions of high tension.^[^ ^20^ ^]^ Of note, the Arp2/3 complex may orchestrate the above responses through its interactions with nucleation‐promoting factors such as Wiskott–Aldrich syndrome protein (WASP) family members, thereby harmonizing cytoskeletal dynamic under forces.^[^ ^265^ ^]^ Third, mechanical forces may increase the density and geometry of F‐actin without changing actin composition,^[^ ^266^ ^]^ indicating the network‐level control of mechanical forces on F‐actin dynamics.^[^ ^252^ ^]^

F‐actin mechanotransduction may also participate in the nucleo‐plasmic transportation of YAP/TAZ and MRTF/SRF. Increased cytoskeletal tension, e.g., enhanced distribution of tensed actin‐cap fibers between the peripheral versus perinuclear adhesions,^[^ ^267^ ^]^ can promote nuclear shuttling of YAP/TAZ, which is hindered by reduced F‐actin density.^[^ ^145^ ^]^ Additionally, force‐induced actin polymerization may facilitate the release and subsequent nuclear shuttling of MRTF, as cytoplasmic individual G‐actins that sequester MRTF are diminishing.^[^ ^134^, ^268^ ^]^ Shuttling of YAP/TAZ^[^ ^147^ ^]^ and MRTF/SRF^[^ ^268^ ^]^ regulate the expression of genes encoding cytoskeletal dynamics,^[^ ^115^ ^]^ forming a long‐term feedback loop involving mechanotransduction and gene expression.

#### 7.4.2 Other Cytoskeletons

Microtubule mechanotransduction is also essential for cellular function and division.^[^ ^101^, ^269^ ^]^ Releasing compressive force at the microtubule end is thought to induce tubulin (microtubule's monomer) polymerization,^[^ ^29^ ^]^ thereby linking the mechanical forces of polymers to the chemical potential of monomers.^[^ ^269^ ^]^ By contrast, increasing compressive forces stabilize microtubules in living cells, which is instrumental for maintaining cell shape and migration in confined space.^[^ ^270^ ^]^ Additionally, stiff substrates, increased F‐actin contractility, and FA expansion enhance microtubule acetylation,^[^ ^101^ ^]^ whereas a temporally stiffening substrate leads to microtubule deacetylation.^[^ ^112^ ^]^ Recent studies also indicate the involvement of spectrin,^[^ ^271^ ^]^ keratin, and vimentin cytoskeletons^[^ ^272^ ^]^ in cellular mechanotransduction.

Arguably, cytoskeleton plays a pivotal role in cellular and tissue mechanotransduction. Cytoskeleton is central for regulating the distribution and morphology of organelles,^[^ ^273^ ^]^ cell tensegrity,^[^ ^30^ ^]^ mechanics,^[^ ^254^ ^]^ and behaviors,^[^ ^171^ ^]^ as well as tissue mechanical status.^[^ ^274^ ^]^ Furthermore, cytoskeleton mechanotransduction is capable of producing various biochemical and functional responses at the cellular and tissue levels. Correspondingly, modulating cytoskeletal organization and function could affect mechanical stimuli and biochemical responses. Hence, a deeper understanding of cytoskeletal mechanotransduction will be essential to gain the complete picture of cellular mechanotransduction.

### 7.5 Nuclear Mechanotransduction

In nuclear mechanotransduction, mechanical stimuli can transmit along a structural routine to relay to nuclear surface^[^ ^33^ ^]^ and interior,^[^ ^198^, ^275^ ^]^ (see the next Box 1), and induce responses converging on the spatiotemporal regulations on multidimensional arrangement of nuclear genome and thus gene activities.^[^ ^276^ ^]^

1 **Box 1. Structural routine of forces relaying to nuclear surface and interior**

Nuclear mechanotransduction is building a detailed routine of forces relaying to nuclear surface and interior. Nucleus uses the ‘Linker of the Nucleoskeleton and Cytoskeleton’ (LINC)^[^ ^198^, ^275^ ^]^ to connect its inner components with specialized perinuclear cytoskeleton.^[^ ^267^ ^]^ Specifically, on outer nuclear membrane (ONM), the nesprins of LINC interact with all three cytoskeletal filaments in cytoplasm. Meanwhile, on inner nuclear membrane (INM), the SUN proteins of LINC bind with the nuclear lamina, NPCs and chromatins in nucleoplasm [4–6].^[^ ^277^, ^278^, ^279^ ^]^ Moreover, nuclear lamina, consisting of lamins (lamin A/C or lamin B, encoded by LMNA A/B gene), especially lamin A/C, anchors chromatin to nuclear envelope (NE) through binding with the lamina‐associated domains (LADs) of chromatin.^[^ ^280^ ^]^ In addition, NPCs span NE to transport molecules between the nucleus and cytoplasm^[^ ^281^ ^]^ and structurally associate with LINC,^[^ ^278^ ^]^ nuclear lamina,^[^ ^282^ ^]^ and cytoskeleton. As well, an INM protein, emerin, also concurrently connects to the lamin A/C and chromatin.^[^ ^283^ ^]^

At the molecular level, lamin A/C, emerin, and chromatin can sense mechanical forces through different mechanisms. Forces may alter lamin A/C conformation and reduce their phosphorylation and density,^[^ ^204^ ^]^ resulting in changes in nuclear integrity and stiffness,^[^ ^284^ ^]^ chromosome distribution,^[^ ^280^ ^]^ epigenetic state,^[^ ^157^ ^]^ and transcriptional activity.^[^ ^280^ ^]^ Inversely, mechanical stretch increases the phosphorylation of emerin^[^ ^283^ ^]^ and facilitates their translocation to outer nuclear membrane (ONM),^[^ ^285^ ^]^ thereby empowering perinuclear actin polymerization, changing chromatin organization, and regulating gene expression. Notably, forces also directly affect the conformation, gene accessibility, location, and mechanic of chromatin.^[^ ^285^, ^286^ ^]^ Interestingly, forces applied on the interphase chromatin, even as minor as those forces repositioning intranuclear molecules, can easily relocate genomic loci across nucleus in minutes, indicating that interphase chromatins are more force‐sensitive than previously understood.^[^ ^287^ ^]^

Mechanical forces may also have a role in the sub‐nuclear features of 3D genomic organization.^[^ ^117^, ^288^ ^]^ As the sub‐nuclear 3D structures, chromosome intermingling regions are enriched with active RNA polymerase II, transcription factors, histone modifiers,^[^ ^289^ ^]^ and genes that are spatially clustered and co‐regulated, providing a factory for gene expression.^[^ ^241^ ^]^ It has been noted that adequate mechanical forces can disrupt the intact physical associations required for establishing the co‐transcriptional hubs in chromosome intermingling,^[^ ^290^ ^]^ and thus reprogram the 3D configuration and gene expression.^[^ ^241^ ^]^ Additionally, liquid‐liquid phase separation (LLPS) which underpins the formation of biomolecular condensates, has a crucial role in regulating chromation organization and gene expression.^[^ ^291^, ^292^ ^]^ It was found that the surface tension of liquid droplets is an essential modulator of nuclear LLPS,^[^ ^288^ ^]^ indicating another possible interaction between mechanical forces and nuclear gene expression, despite of unclear mechanisms.

During cellular processes, force‐induced changes in nuclear morphology^[^ ^117^ ^]^ could impact the positions of chromosomes,^[^ ^293^ ^]^ epigenetic states of chromosomes, and transcriptional activity of specific genes,^[^ ^294^ ^]^ highlighting a mechanotransduction mechanism at the whole‐nuclear level. Chromosome position is more likely to be altered by fluctuant tension of nuclear envelop (NE) rather than other minor mechanical perturbances.^[^ ^293^, ^294^ ^]^ In alignment with this, heterochromatins that are dense and transcriptionally inactive are close to the nuclear periphery, whereas euchromatins that are loose and transcriptionally active localize at the nuclear interior.^[^ ^295^ ^]^

Recent evidence suggests an involvement of other mechanisms in nuclear mechanotransduction. For example, the nucleocytoplasmic shuttling of epigenetic factors may mediate the force‐induced epigenetic changes.^[^ ^296^, ^297^ ^]^ Excessive forces could rupture NE, leading to uncontrolled exchange of cytoplasmic and nuclear proteins,^[^ ^298^ ^]^ organelle mislocalization,^[^ ^299^ ^]^ DNA damage,^[^ ^298^ ^]^ and altered gene expression.^[^ ^300^ ^]^ Furthermore, enhanced NE tension upregulates the opening of channels, facilitating nucleo‐plasm transport of proteins/ions and activating Ca^2+^‐dependent phospholipase (cPLA2).^[^ ^113^, ^301^ ^]^ In fact, these nuclear mechanotransduction pathways at different scales are collectively and coordinatively responsible for modifying gene landscape and activities. Furthermore, building on the nuclear mechanotransduction, cell may tailor a mechanogenomic code that establishes the correlations between genomic landscape and mechanical context.^[^ ^117^, ^241^ ^]^

### 7.6 Other Molecular and Subcellular Mechanotransduction

Other subcellular structures have emerging roles in mechanotransduction. For example, enhanced membrane tension can function in a way independent of actin and ATP^[^ ^302^ ^]^ to diminish the interactions between caveolin/caveolin1 and disassemble/flatten caveolae, leading to increased free caveolins and reduced membrane tension.^[^ ^158^ ^]^ Nonetheless, caveolin1 also modifies membrane curvature independently of caveolae, which confers cells with deformability and mechanoprotection.^[^ ^303^ ^]^ Mechanical forces can change the conformations of G protein‐coupled receptors (GPCRs)^[^ ^304^, ^305^ ^]^ and further activate downstream proteins,^[^ ^306^ ^]^ possibly via the helix 8 motif in GPCR.^[^ ^307^ ^]^ In terms of the latent‐TGF‐β‐binding protein (LTBP)‐latency‐associated peptide (LAP)‐TGF‐β complex in ECM,^[^ ^199^ ^]^ intracellular forces can transmit along integrins to change LAP conformation, thereby releasing mature TGF‐β to regulate cell behaviors. Additionally, forces are capable of triggering the catch‐bond behaviors of molecules at cell‐cell interfaces, including glycocalyx,^[^ ^308^ ^]^ peptide/major histocompatibility complexes‐T cell receptor (pMHC‐TCR),^[^ ^309^ ^]^ Notch ligands,^[^ ^310^ ^]^ and MHC class I‐LILRB3.^[^ ^311^ ^]^ Functionally, they result in local molecular contexts different from the rest plasma membrane and thus prime downstream signals.^[^ ^242^, ^312^ ^]^

**Figure 7:**
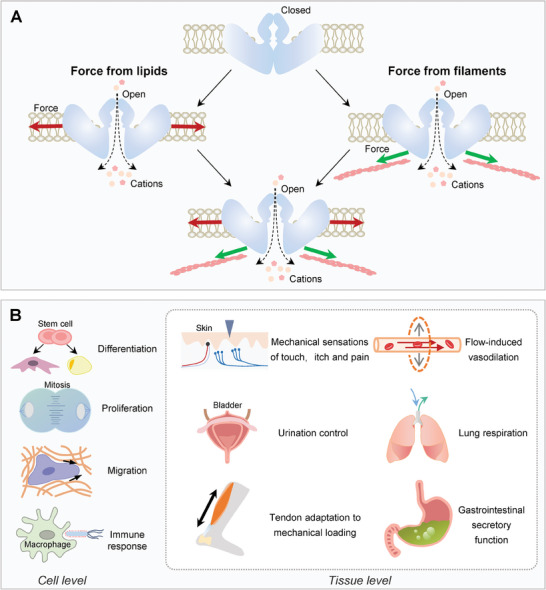
Mechanosensitive mechanism and function of Piezos. A) Piezo channels are nonselective cationic mechanosensitive channels that are permeable to alkali ions, divalent cations, and organic cations. The “force from lipids” model signifies that the forces from the lipid bilayer open the Piezos by creating curvatures, while the “force from filaments” model implies that the cytoskeletal forces change the conformations of Piezos. The mixed model emphasizes the complementary roles of both the membrane tension and cytoskeletal forces in opening Piezos. B) Mammalian Piezos are expressed in multiple organs and tissues, and essential for transducing externally and internally applied forces at the plasma membrane. Piezos can activate downstream signals and contribute to the diverse set of roles both at the cell and tissue level.

Some ion channels such as Piezo1/2, transient receptor potential vanilloid 4 (TRPV4), TRPC, TACAN, and SWELL1, can transform their conformations under forces to facilitate ion influx and intracellular biochemical signals.^[^ ^313^ ^]^ Particularly, Piezo1/2 are gaining the most investigations due to their versatile roles in many processes,^[^ ^206^ ^]^ including immune response,^[^ ^176^ ^]^ touch sensation,^[^ ^314^ ^]^ cognitive functions,^[^ ^315^ ^]^ gastrointestinal movements,^[^ ^316^ ^]^ tendon adaptation to physical burden,^[^ ^317^ ^]^ flow‐induced vasodilation,^[^ ^91^ ^]^ urination control of bladder,^[^ ^318^ ^]^ and antigenic function of erythrocyte^[^ ^319^ ^]^ (**Figure** **7**). Mechanistically, it is now increasingly approved that forces from plasma membrane may directly open the Piezo 1 channel,^[^ ^320^, ^321^ ^]^ despite that forces from cytoskeleton and adhesions might contribute to this process.^[^ ^322^ ^]^

Remarkably, biomolecular condensate is increasingly viewed as a mechano‐regulator of certain signaling pathways. Specifically, spontaneous PI(4,5)P2 phase separation (a lipid phase separation), initiated by reduced plasm membrane tension, is sufficient to induce the clustering and inactivation of mTORC2.^[^ ^323^ ^]^ Increased substrate stiffness triggers the receptor tyrosine kinase discoidin domain receptor 1 (DDR1) to form biomolecular condensates with the large tumor suppressor kinase 1 (LATS1), thereby inactivating LATS1 but activating YAP.^[^ ^324^ ^]^ These findings, together with the associations between forces and LLPS^[^ ^325^ ^]^ and the role of biomolecular condensates in nuclear and cytoskeletal functions,^[^ ^326^ ^]^ suggest an entangled interplay between biomolecular condensates and mechanotransduction.

## 8 Mechanotransduction in Diseases

**Figure 8:**
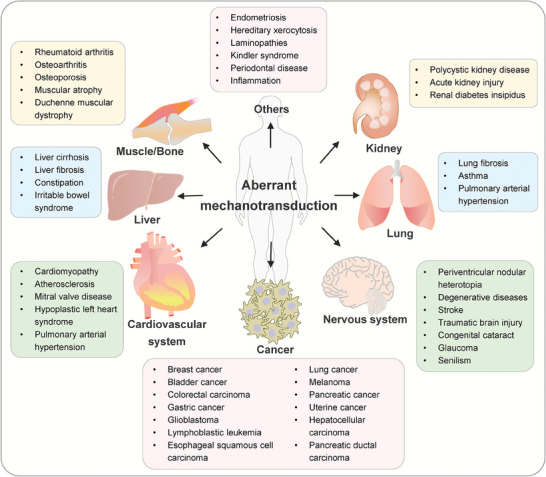
Diseases related to aberrant mechanotransduction. Under normal conditions, physiological mechanical stimulation have a critical impact on the sustainment of healthy, whereas continuous excessive mechanical stimulation could contribute to a wide range of pathologic conditions and diseases of multiple organs. Therefore, mechanotransduction signaling cascades may be potential therapeutic targets for clinical treatment. However, the precise mechanisms between mechanical cues and diseases are still not fully elucidated and remain challenging. The online resource inside this figure was quoted or modified from Scienceslide2016 plug‐in.

As mentioned above, mechanotransduction plays essential roles in establishing and retaining a mechanical, biochemical, and biological homeostasis, and thus aberrant mechanobiology could cause multiple diseases, ranging from cardiovascular diseases, nervous system diseases, digestive system diseases to various cancers (**Figure** **8**).

### 8.1 Cardiovascular Diseases

**Figure 9:**
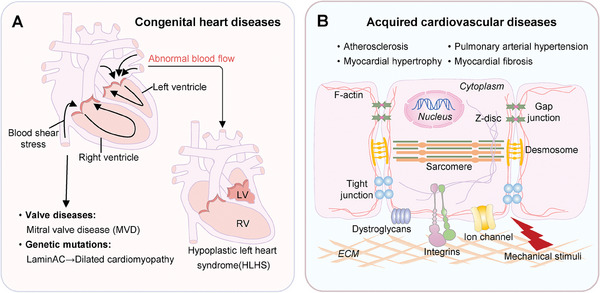
Schematic of cardiovascular diseases caused by mechanotransduction. A) Mechanical stimuli plays an important role in the regulation of embryonic heart development. Mechanoreceptors on the surface of endocardial cells and cardiomyocytes regulate gene expression in response to blood flow. It has shown that abnormal blood shear stress during embryonic development could contribute to cardiac dysplasia, such as hypoplastic left heart syndrome (HLHS) and valvular diseases. B) When adult heart is under prolonged and abnormal loading conditions, it might become maladaptive, leading to a series of cardiac diseases. Either abnormal mechanical stimuli or impairment of the heart's mechanotransduction processes could lead to acquired cardiovascular disease, such as atherosclerosis, myocardial fibrosis, myocardial hypertrophy, and pulmonary arterial hypertension.

Consistent with the prominent role of mechanobiology in cardiovascular physiology, cardiovascular diseases, including the congenital and acquired ones (**Figure** **9**), can be instigated by inappropriate mechanical stimuli and mutations of genes associated with mechanotransduction. Abnormal blood flow at early embryonic stages could cause hypoplastic left heart syndrome (HLHS), one of the classic congenital cardiac defects.^[^ ^327^ ^]^ Similarly, in the pathogenesis of hypertrophic cardiomyopathy, addition in the amount and volume of sarcomere caused by gene mutations could increase cardiac wall thickness, disrupt cardiac mechanics, and cause a smaller space blocking blood flow.^[^ ^328^ ^]^ Mutations in *LMNA* gene that lead to the mislocalization or abnormal expression of emerin^[^ ^329^ ^]^ and Sad1 and UNC84 domain containing 1 (SUN1)^[^ ^330^ ^]^ could result in laminopathies, such as dilated cardiomyopathy (DCM).^[^ ^331^ ^]^

Acquired cardiovascular diseases, including atherosclerosis^[^ ^332^, ^333^ ^]^ and hypertension,^[^ ^177^ ^]^ are aggravated by the disturbances in hemodynamic forces and tissue stiffness. In the deterioration of atherosclerosis, abnormal shear stress provokes the progression of unstable plaques by inducing pathological consequences, including inflammation, surplus proliferation and apoptosis of endothelial cells, endothelial‐to‐mesenchymal transition (EndoMT), accumulation of lipid proteins, and ECM remodeling.^[^ ^86^ ^]^ These changes are partially attributed to abnormal shear stress‐triggered activation of signaling pathways, such as nuclear factor kappa‐B (NF‐κB), TGF‐β, YAP/TAZ, phosphatidylinositol‐3 kinase (PI3K)/AKT, ALK5‐Shc,^[^ ^334^ ^]^ and epigenetical induction of the expression of NF‐κB and homeobox family members.^[^ ^335^ ^]^ Of note, recent studies have highlighted the contribution of classic mechanotransducers,^[^ ^336^ ^]^ including the mechanosensitive channels, GPCRs, integrin‐adhesions, CAs, caveolae, cytoskeleton and nucleus, as well as their feedback loops, to pathological remodeling concerning atherosclerosis.^[^ ^333^ ^]^

Different from physiological cardiac hypertrophy driven by hemodynamic overload, pathological hypertrophy caused by chronic hypertension is associated with maladaptive ECM remodeling, myofibroblast activation, cardiomyocyte death, and re‐activated fetal genes.^[^ ^337^, ^338^, ^339^ ^]^ These pathological changes are caused by impaired Ca^2+^ and reactive oxygen species (ROS) homeostasis, hyperactive signaling pathways (e.g., mTORC2/AKT, ERK, and YAP), and altered transcription circuits,^[^ ^340^ ^]^ which may be predominantly mediated by mechanotransduction encompassing mechanosensitive channels,^[^ ^339^, ^341^ ^]^ caveolae, adhesions, and specialized structures in cardiomyocyte (e.g., sarcomere, desmosomes, and intercalated disc).^[^ ^342^ ^]^ In this regard, targeting mechanotransduction could be a valuable strategy to treat patients affected by this devastating disease.

### 8.2 Cancers

**Figure 10:**
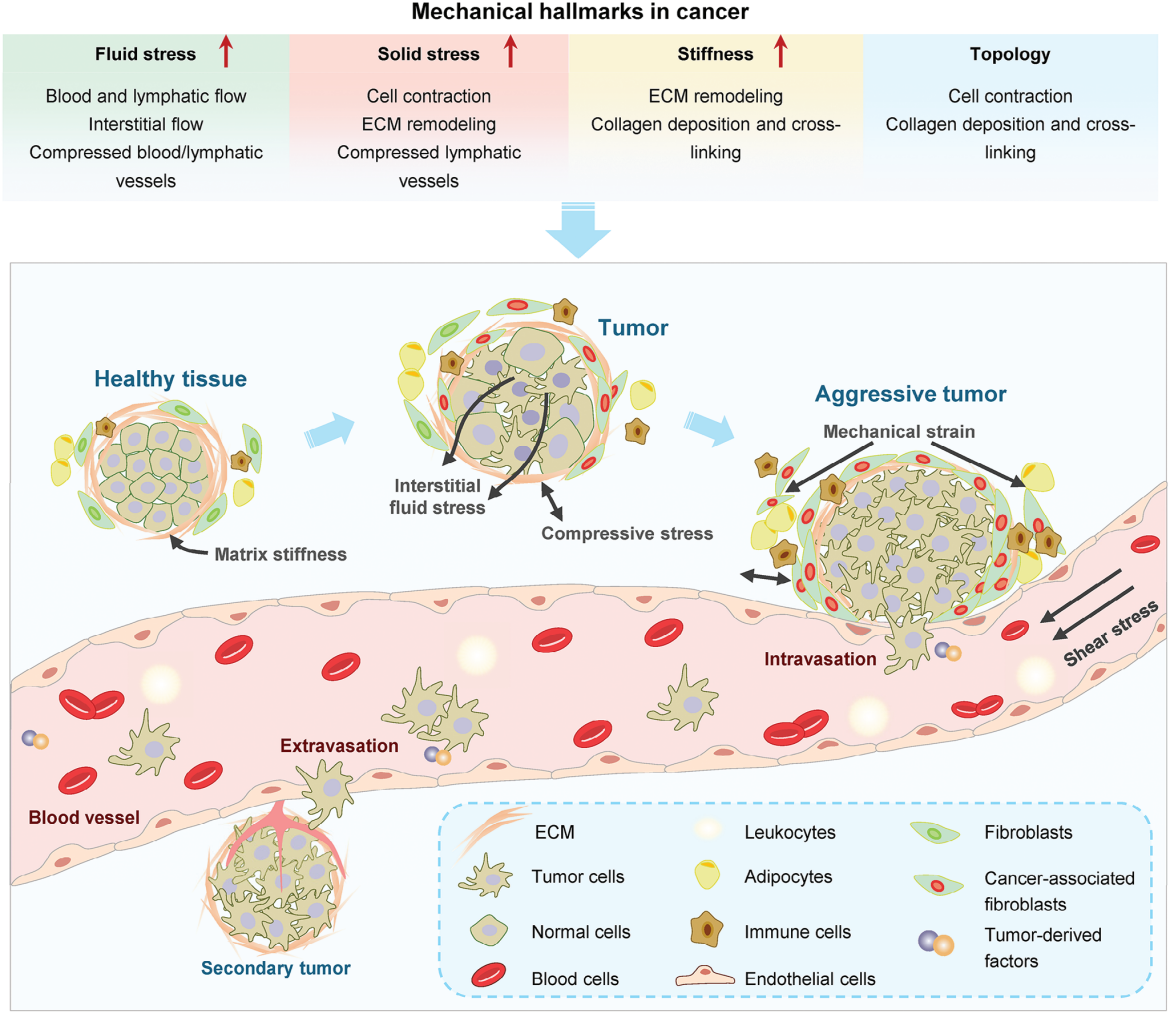
Mechanotransduction in tumor progression. In tumorigenesis, cells undergo a variety of mechanical stimuli from the surrounding microenvironment and adjacent cells. Meanwhile, tumor cells can generate internal high‐pressure solid stress, resulting in increased blood/interstitial pressure. Consistent with this, uncontrolled proliferation of tumor cells constantly presses adjacent tissues and tumor vasculature to cause abnormal tumor vasculature. Additionally, the multiple stromal cell types, such as cancer‐associated fibroblasts, shape the tumor microenvironment. The ECM stiffens and arranges themselves in an anisotropic orientation due to the activity of the stromal cells, which facilitate the invasion of tumor cells towards the blood circulatory system, ultimately colonizing in secondary niches, such as the lung, bone, and brain.

Mechanotransduction has been extensively implicated in many tumor progression procedures, including expansion, migration, metastasis, drug resistance, angiogenesis, and immune evasion^[^ ^343^ ^]^ (**Figure** **10**). Central to understanding this is the appreciation of two aspects: first, tumor microenvironment (TME) is full of mechanical stimuli, including solid stress, fluid stress, substrate stiffness and topological architectures, which are usually oscillated and curial for tumor progression;^[^ ^343^ ^]^ second, numerous oncogenic factors have the potential to alter the mechanical properties of cells and tissues as well as their ability to sense mechanical stimuli.^[^ ^344^, ^345^ ^]^

#### 8.2.1 Cancer mechanotransduction

Expansive tumors in confined contexts usually experience elevated surrounding solid stresses,^[^ ^343^ ^]^ which may elicit various mechanotransduction pathways and affect diverse tumor behaviors. Increased solid stress can damage blood vessels to disturb the supply of nutrient and oxygen and cause hypoxia state,^[^ ^346^ ^]^ conferring tumors with metastatic ability, resistance to immune response and therapy,^[^ ^347^ ^]^ and surviving ability in cruel context.^[^ ^348^ ^]^ Moreover, solid stress may modify genomic states by deforming nucleus, thus benefiting the invasion and metastasis of tumor cells in a long duration.^[^ ^349^ ^]^ Additionally, given the frequency of positive feedback among mechanotransduction pathways, increased solid stress might considerably contribute to the enlarged contractility of tumor cells and ECM remodeling.^[^ ^343^ ^]^

Increasing ECM stiffness in tumors could nurture many malignant phenotypes and facets by altering signaling pathways and gene expression.^[^ ^350^ ^]^ High ECM stiffness can activate the YAP/TAZ signaling to upgrade proliferation, repress anoikis, and increase stemness of tumor cells.^[^ ^351^ ^]^ It is also sufficient to increase intracellular tension that mightily reprograms mitochondrial and metabolic states,^[^ ^174^ ^]^ warranting the tremendous energy requirement for metastasis and other energy‐consuming activities.^[^ ^352^ ^]^ These changes have shown to be conducive to rapid proliferation, enhanced invasion, and metastasis of several tumors, such as pancreatic ductal carcinoma, hepatocellular carcinoma,^[^ ^353^ ^]^ breast cancer,^[^ ^354^ ^]^ and esophageal squamous cell carcinoma.^[^ ^355^ ^]^ Moreover, high ECM stiffness can abolish vascular integrity and drive angiogenesis^[^ ^356^ ^]^ through activating PI3K/AKT^[^ ^357^ ^]^ and FAK/Src pathways.^[^ ^358^ ^]^ Instead, a soft ECM enhances vascular integrity to raise tumor susceptibility to chemotherapy and radiotherapy.^[^ ^359^, ^360^ ^]^

Liquid leaking from impaired blood and lymphatic vessels can cause anisotropic interstitial fluid pressures throughout the tumor. Multiple mechanotransducers sense these interstitial fluid pressures and trigger various biochemical signals concerning tumor immunity, fibroblast transformation,^[^ ^361^ ^]^ angiogenesis/lymphangiogenesis,^[^ ^362^ ^]^ matrix metalloproteinase (MMP) activity,^[^ ^363^ ^]^ migration,^[^ ^364^ ^]^ and proliferation.^[^ ^365^ ^]^ Besides, progressive tumors often experience alterations in cell shape,^[^ ^366^ ^]^ context dimensionality,^[^ ^367^ ^]^ and local tissue architecture,^[^ ^368^ ^]^ which act, both interdependently and coordinately, through multiple mechanotransduction pathways, e.g., adhesion‐mediated signaling, Pizeo‐mediated Ca^2+^ signaling, and nucleus‐mediated genomic changes,^[^ ^369^ ^]^ to modulate the migration, epithelial‐mesenchymal transition (EMT),^[^ ^343^ ^]^ and malignancy of tumor cells.^[^ ^349^ ^]^

Intriguingly, oncogenes have been found to widely alter the mechanical properties and sensitivity of tumor cells and tissues, establishing vicious cycles of biochemical and mechanical changes in tumor progression. For example, Ras, the most frequently mutated oncogene, has a strong ability to change cell contractility and adhesion, leading to altered tissue mechanics and cellular sensitivity to the surrounding stiffness.^[^ ^344^, ^370^, ^371^ ^]^ Moreover, recent data indicate that Ras activation is associated with the mechanoresponsive YAP/TAZ pathway, complicating the manifold layers between tumor progression and mechanotransduction.^[^ ^344^ ^]^

#### 8.2.2 Cross‐Talk between Mechanotransduction and Cancer Hallmarks

**Figure 11:**
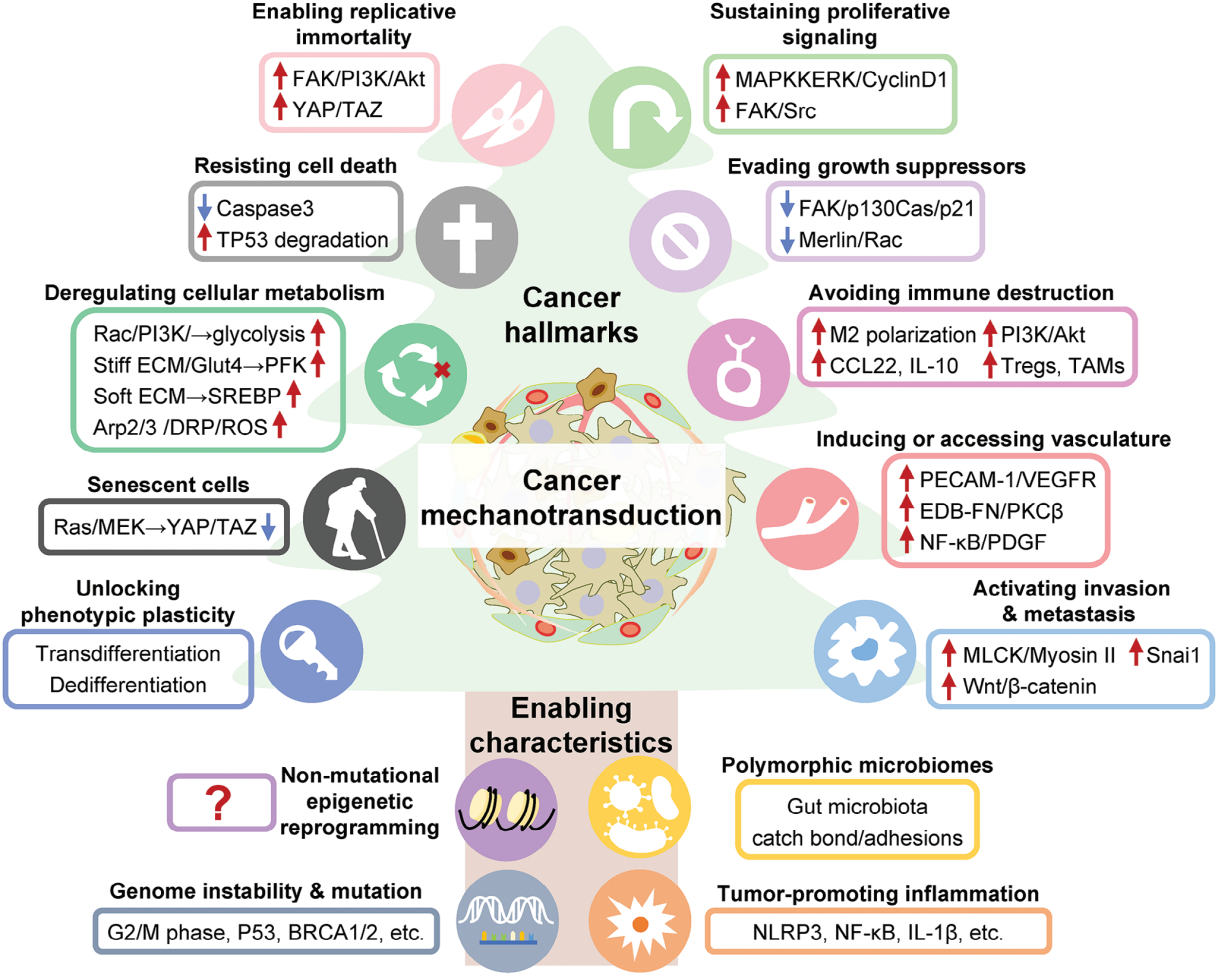
Cross‐talk between mechanotransduction and hallmarks of cancer. An increasing body of research suggests that mechanotransduction is involved in the pathogenesis of some and perhaps all cancers, and closely associated with the hallmarks of cancer, including ten basic hallmarks (Upper part of the tree) and four enabling characteristics (Root of the tree). This diagram shows several key pathways or processes of mechanotransduction that are involved in tumorigenesis. Interestingly, these signaling pathways may also interact with each other to form an intricate ‘mechanobiology‐cancer network’, collectively promoting tumor initiation and progression.

Since mechanotransduction can influence almost every process of tumorigenesis, exploring the reasons for the continuous “interplay” between mechanobiology and cancer biology may advance this field. Recently, Dr. Douglas Hanahan has enumerated and updated hallmarks of cancer^[^ ^372^ ^]^ that include ten basic hallmarks and four enabling characteristics, all of which are nearly associated with mechanotransduction (**Figure** **11**). For instance, mechanical stresses within tumors could promote the mesenchymal‐like transdifferentiation of cells,^[^ ^373^ ^]^ which increases the phenotypic plasticity and induces a stem‐like phenotype to further facilitate tumorigenesis. Declining YAP/TAZ mechanotransduction has been raised to drive cell senescence by unleashing the cGAS‐STING signal.^[^ ^374^ ^]^ Of note, the impact between mechanotransduction and nonmutational epigenetic reprogramming is currently debated. Emerging evidence has proposed that cell could sense and respond to the surroundings by transmitting mechanical stimuli into biochemical signals, and thus affect epigenetic states,^[^ ^375^ ^]^ such as DNA/histone methylation,^[^ ^376^ ^]^ histone acetylation, and microRNA expression,^[^ ^377^ ^]^ whose dysregulation are involved in disease progression.^[^ ^378^, ^379^, ^380^ ^]^ We believe that an increasing number of prospective studies will fill the gap between mechanotransduction and non‐mutational epigenetics. Collectively, an in‐depth study of the relationship between the integrative concept of cancer hallmarks and mechanobiology may provide opportunities for the discovery of cancer medicine and therapy strategies.

### 8.3 Musculoskeletal Diseases

Abnormalities in mechanical stress or mutations in mechanotransduction‐related molecules would lead to skeletal muscle diseases, such as osteoporosis, arthritis, and muscular dystrophy. As bone is highly sensitive to changes of daily mechanical loading and gravity, lack of mechanical stimuli, such as long‐term weightlessness or paralysis, may reduce bone mass and increase the risk of fractures.^[^ ^381^ ^]^ Recently, it has been reported that several FA‐related molecules have critical roles in regulating bone microenvironment and homeostasis through mechanotransduction,^[^ ^382^, ^383^ ^]^ indicating a potential therapeutic way for metabolic bone diseases. In addition, high mechanical stress has been implicated in aggravating inflammatory arthritis, including spondyloarthritis and rheumatoid arthritis.^[^ ^384^ ^]^ Intriguingly, absence of dystrophin can disorder microtubule cytoskeleton, which increases X‐ROS and disrupts Ca^2+^ homeostasis, ultimately contributing to the fatal X‐linked degenerative muscle disease called Duchene muscular dystrophy (DMD).^[^ ^385^, ^386^ ^]^ Given the significance of muscle in sensing, transmitting, and producing mechanical forces, an increasing involvement of mechanotransduction in musculoskeletal diseases is highly anticipated in the near future.

### 8.4 Metabolic Disorders

Accumulating evidence suggests important pathological roles of dysregulated mechanotransduction in metabolic diseases, including nonalcoholic fatty liver disease (NAFLD) and type 2 diabetes mellitus (T2DM). For example, the inactivation of CapZ resulted in liver tissue stiffening, and in return activated the YAP/TAZ to inhibit gluconeogenic enzymes, ultimately leading to liver metabolic defects.^[^ ^387^ ^]^ In T2DM, high glucose concentration is able to induce the osmotic swelling of islet β cells, increasing membrane tension to activate mechanosensitive ion channels that impair insulin secretion.^[^ ^180^, ^388^ ^]^ Interestingly, SWELL, an ion channel controlling insulin sensitivity, is of mechanosensitivity, suggesting a link of mechanotransduction to insulin resistance,^[^ ^172^ ^]^ the key pathological change in various metabolic disorders which is also regulated by numerous molecules.^[^ ^389^, ^390^, ^391^, ^392^ ^]^ In addition, hyperglycemia can alter the mechanical properties of plasm membrane of dorsal root ganglion neurons by eliciting osmotic disorder, methylglyoxal and ROS overproduction, therefore bringing about the malfunction of mechanosensitive channels and thus diabetic neuropathy progression.^[^ ^393^ ^]^

### 8.5 Inflammation and Fibrosis

There are notable linkages between mechanotransduction and inflammation,^[^ ^394^ ^]^ which further contribute to tissue fibrosis by stiffening ECM macroscopically. On the one hand, aberrant mechanical stimuli could affect inflammation by controlling leukocyte recruitment, fibrogenic response, and ECM remodeling.^[^ ^395^ ^]^ For example, combined forces from intercellular cell adhesion molecule‐1 (ICAM‐1) clustering and blood shear increase endothelial plasma membrane tension, subsequently activating Piezo and downstream signaling to initiate leukocyte diapedesis.^[^ ^396^, ^397^ ^]^ On the other hand, inflammatory state may impact the mechanical context and mechanotransduction. For instance, excessive ECM deposition resulting from chronic inflammation facilitated the activation of β‐catenin signaling,^[^ ^398^ ^]^ and further influenced the progression of fibrosis through hyperactivated Rho/ROCK, YAP/TAZ, and other FAK‐mediated pathways.^[^ ^399^, ^400^ ^]^ Subsequently, when fibrosis is out of normal control, it would develop to pathological scarring, such as skin keloids.^[^ ^401^ ^]^

## 9 Mechanotherapy

**Figure 12:**
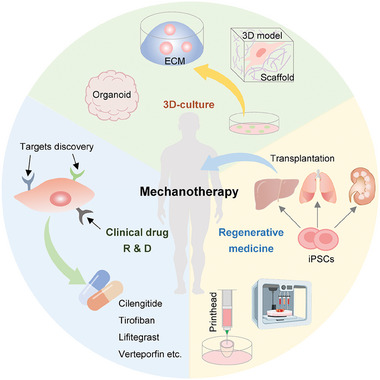
Emerging applications of mechanotransduction. Mechanotransduction has been applied in many aspects, including clinical drug discovery, 3D‐culture (microenvironment simulation, and organoid construction), and regenerative medicine (tissue regeneration, and 3D/4D printing). The interdisciplinary of physics, biomechanics, biomaterials, etc., could further promote the transformation from basic disciplines to clinical practice, ultimately promoting the development of mechanotherapy and benefitting human health. The online resource inside this figure was quoted or modified from Scienceslide2016 plug‐in.

Mechanical cues and mechanotransduction are increasingly considered as a potential toolbox for applications spreading from basic studies on cell biology to clinical transformation (**Figure** **12**). Recently, the employment of physical means to clinical treatment has attracted interest and thus the term mechanotherapy was proposed.^[^ ^402^ ^]^ Mechanotherapy, defined as “therapeutic interventions that reduce and reverse injury to damaged tissues or promote the homeostasis of healthy tissues by mechanical means at the molecular, cellular, or tissue level,”^[^ ^403^ ^]^ is a groundbreaking approach by integrating several disciplines, including biochemistry, bioinformatics, biomaterials, tissue engineering, and regenerative medicine.

### 9.1 Clinical Drug Development

Mechanical cues could affect the response to drugs by influencing the cellular programs and states, which have a two‐sided impact on the development of clinical drugs. On the one hand, the importance of mechanical stimuli and mechanotransduction in controlling signaling pathways (Figure 5) is enabling mechanotransduction as a target for medicine discovery, aiding rapid development of drugs (**Table** **1**). For example, Lifitegrast, an integrin antagonist, can suppress the T cell‐mediated inflammation and has been approved for treatment of dry eye disease (DED).^[^ ^404^ ^]^ Besides, therapeutics that directly modulate the mechanical TME have demonstrated remarkable clinical efficacy for increasing tumor perfusion and drug delivery,^[^ ^405^ ^]^ through reducing tumor stiffness and mechanical forces with administrating pirfenidone, tranilast, and dexamethasone.^[^ ^406^ ^]^ On the other hand, aberrant actions of mechanotransduction components are possible to drive resistance to drugs or chemotherapy. For example, abnormally activated integrins, due to changes in the TME, impaired drug action by promoting anti‐apoptotic protein expression, blocking cell cycle, and facilitating metastatic reactivation, ultimately leading to drug and chemotherapy resistance.^[^ ^407^, ^408^ ^]^ Similarly, improper internal forces, such as enhanced ECM stiffness and sheer stress, could hinder drug delivery. Consistently, many solid tumors exhibit poor responses to drugs following abnormal shear stress.^[^ ^409^, ^410^ ^]^ Therefore, an in‐depth study of the mechanisms between mechanotransduction and drug resistance will improve the efficacy of targeted therapies remarkably.

**Table 1:** Drugs targeting mechanotransduction.

Agent	Molecular target	Indication	Phase	NCT number	Reference
Cilengitide	Integrin (*α_v_β_3_, α_v_β_5_*)	NSCLC	I	NCT01118676	[450]
		Glioma	III	NCT00689221	[451]
PF00562271	FAK	Pancreatic neoplasm	I	NCT00666926	[452, 453]
GSK2256098	FAK	Hypertension, pulmonary	I	NCT02551653	[404, 454]
Simtuzumab	LOXL2	Liver fibrosis/cirrhosis	II	NCT01672853	[452, 455]
CK‐1827452	Myosin II	Heart failure	II	NCT00748579	[456]
SB‐705498	TRPV1	Rhinitis	II	NCT01476098	[454, 457]
GSK2798745	TRPV4	Heart failure	II	NCT02497937	[458, 459]
Defactinib	FAK	MPM	II	NCT01870609	[459]
Belumosudil	ROCK	Psoriasis vulgaris	II	NCT02317627	[460]
Fasudil	ROCK	Raynaud	III	NCT00498615	‐
ML‐7 HCl	MLCK	HIV infection	III	NCT00190242	[457, 458, 461]
Verteporfin	YAP/TEAD	Age‐related macular degeneration	IV	NCT01846273	[453]
Tirofiban	Integrin (*α_v_β_3_)*	NSTEMI	IV	NCT03114995	[404]
Lifitegrast	Integrin	DED	IV	NCT03451396	[462]
Nintedanib	VEGFR	Idiopathic pulmonary fibrosis	IV	NCT02598193	[450]
Triolein	MMP‐1	Obesity	‐	NCT01128400	‐

NSCLC, non‐small cell lung cancer; MPM, malignant pleural mesothelioma; NSTEMI, non‐ST‐elevation myocardial infarction; DED, dry eye disease; HIV, human immunodeficiency virus.

Of note, mechanotransduction is strictly and dynamically controlled by several highly homologens protein families, such as integrins, which represents a common challenge in developing drugs for mechanotransduction. For example, the RGD‐binding integrins α_v_β_6_ and α_v_β_8_ are considerable therapeutic targets for cancer and fibrosis; however, there is no compound that can effectively distinguish them. Recently, Roy et al. developed a structure‐based de novo design strategy and successfully obtained selective and potent α_v_β_6_ and α_v_β_8_ inhibitors.^[^ ^411^ ^]^ As exemplified by this approach, it is reasonable that the advance of similar strategies would throw insights into mechanotransduction‐based drug discovery.

### 9.2 3D‐Culture: Microenvironment Simulation and Organoid Fabrication

Although the 2D‐culture system is widely used, it ignores the complexity and heterogeneity of the real tissue microenvironment. The 3D‐culture system is undoubtedly closer to the authentic environment within the body, due to its ability to replicate the biological tissues, microenvironment, and the mechanical signals. Therefore, it is widely believed that 3D‐culture systems, including organoid, could provide powerful platforms for in vitro pathogenesis studies and drug screening.^[^ ^412^ ^]^ Indeed, proper mechanical stimuli and a beneficial 3D microenvironment are important to maintain chondrogenic phenotypes,^[^ ^413^ ^]^ facilitate fibrillar adhesions that are extremely close to fibroblasts in vivo,^[^ ^414^ ^]^ and improve the pluripotency of human embryonic stem cells,^[^ ^415^ ^]^ etc. Notably, 3D‐culture not only needs the consideration of mechanobiology and mechanical parameters, but also relies heavily on biomaterials for technical support.^[^ ^416^, ^417^ ^]^

Currently, 3D brain models based on human induced pluripotent stem cell (hiPSC) neurons are widely used to recapitulate the major phenotypes and metabolic profiles reminiscent of several neurodegenerative diseases, such as Parkinson's disease^[^ ^418^ ^]^ and Alzheimer's disease.^[^ ^419^ ^]^ Within this model, appropriate cell types, mechanical cues, and biomimetic ECM are assembled to mimic physiological and pathological microenvironment of brain. Interestingly, this bioengineered brain model is able to simulate the real gray and white matter respectively to a certain extent.^[^ ^418^ ^]^ Technically, the model is developed based on a porous scaffold composed of fibroin proteins and embedded hydrogels with beneficial mechanical properties, allowing for the formation of functional 3D neural networks. This success suggests that using complex topologies to maximize scaffold surface area and maintaining permeability to the medium may provide a continuously dynamic microenvironment while extending tissue viability simultaneously, ultimately promoting stem cell engineering.

Organoids, acquired from cultured stem cells, can recapitulate essential features of in vivo organ development and regeneration.^[^ ^420^ ^]^ Viable organoid fabrication depends on the establishment of an appropriate mechanical context comprising mechanical forces, mechanical properties, and tissue geometry.^[^ ^421^, ^422^ ^]^ A well‐managed combination of these mechanical stimuli, together with chemical and genetic modifications, holds a greater promise to enhance organoid outcome.^[^ ^421^, ^422^ ^]^ Moreover, due to significant advances in materials, engineering, and computational biology, the creation of highly replicable and controllable organoids that can be used for translational and basic research has made good progress.^[^ ^422^, ^423^ ^]^ For example, decellularized ECM from either natural or synthetic materials is expected to ensure property diversification and adaptation to the culture and growth of diverse organoids.^[^ ^424^ ^]^ In recent years, bioprinted stem cell technology has also made extensive progress, partially attributing to the use of biomechanical clues that could guide the proliferation of stem cells for a variety of complex tissues, including bone, heart, liver, and nerves.^[^ ^425^ ^]^ As this technology becomes more refined and specialized, it might provide a more viable and reliable method for in vitro clinical modeling and in vivo organ transplantation.

Despite these tremendous advances, there are remarkable challenges and limitations concerning 3D‐culture. From a scientific view, the mechanisms of mechanotransduction in 3D microenvironment remain largely unknown. From a technologic view, the physical materials for 3D‐culture should be further optimized, and organoids need to be fabricated more realistically. These improvements would offer extremely critical information for the development of biomaterials that mimic native biophysical properties in the future.

### 9.3 Regenerative Medicine

There have been remarkable advances in the field of regenerative medicine and tissue engineering. However, some important organs, such as the liver, kidney, and heart, are difficult to design due to their complex architectures and limited technology.^[^ ^426^ ^]^ Mechanotransductive principles and proper supportive matrices are expected to play crucial roles in successful regenerative medicine. For example, the regeneration of bone/cartilage relies on the precise modulation of mechanical stimuli, including substrate stiffness, culture dimension, surface curvature, geometrical constraints, and mechanical loading.^[^ ^423^, ^427^ ^]^ Recently invented lacunar hyaluronic acid microcarriers (LHAMCs) could uniquely reshape the ECM through 3D‐culture to induce hyaluronic cartilage microtissue regeneration. In addition, due to the mechanotransductive conditions of LHAMCs, it can also prevent the translocation of β‐catenin to the nucleus and repress chondrocyte dedifferentiation by inhibiting the pathway.^[^ ^428^, ^429^ ^]^

Due to incomplete understanding of vascular structure and technical limitations, the inability to create engineered vascular systems is a perennial problem in regenerative medicine, where rich and complex vascular networks are required to provide nutrients and oxygen to tissues. As mentioned above, mechanotransduction plays an important role in vascular function in various organs, and mechanotransductive principles have vast ramifications for vascular construction and regenerative medicine.^[^ ^430^ ^]^ For example, constant hemodynamic forces acting on endothelial cells could regulate vessel stability and angiogenesis,^[^ ^431^ ^]^ and replicating physiological flow direction can improve endothelial function and more closely resemble the biology of healthy blood vessels.^[^ ^432^ ^]^ Intriguingly, it is reported that a 3D vascular network can be generated by adjusting the mechanical sensitivity, which could further improve the tissue repair ability.^[^ ^433^ ^]^ Meanwhile, the selection of suitable scaffold materials for embedding endothelial cells is critical for the self‐assembly of the vascular network, among which mimicking angiogenic features by synthetic or hybrid materials is indispensable. Excitingly, hyaluronic acid hydrogels can promote vascular differentiation of hiPSCs and the formation of vascular networks through self‐assembly.^[^ ^434^ ^]^

Mechanotransduction may also affect the development of 3D/4D bioprinting.^[^ ^435^, ^436^ ^]^ By leveraging supportive materials and accessible cells, 3D bioprinting could create products with mechanical properties akin to organs including cartilage, bone, and skin, promoting regenerative medicine. Moving a step forward, 4D bioprinting endows the products with the capacity to self‐transform under predetermined stimulation due to reasonable utilization of ‘programmable matter’.^[^ ^437^ ^]^ However, present bioprinting technologies still fail to fully reproduce the morphological, biochemical, and physiological properties of living tissues/organs.^[^ ^438^ ^]^ To address this, some potential solutions are ongoing, such as the application of materials with appropriate stiffness, viscosity, geometry, and ligands, or ECM‐based 3D hydrogel culture systems, the use of perfusable microchannel networks for vascularization, and the addition of biochemical factors,^[^ ^438^ ^]^ exposing a guiding role of mechanotransduction in bioprinting fabrication.^[^ ^439^ ^]^

Together, a variety of promising biomechanical applications regarding mechanotransduction provide powerful support for seeking therapeutic targets, improving 3D‐culture, and building regenerative medicine in the coming decades. These unprecedented advances are greatly thanks to the evolvement in mechanostimulatory materials.^[^ ^440^ ^]^ However, there are some challenges on the path to translational utilizing these biomechanically adaptive materials, such as the acquiring of precise delivering, reliable adhesion, powerful memory, flexible energy supply, and sustained functionality.^[^ ^440^ ^]^ Therefore, an accurate understanding of hierarchical mechanobiology could further promote and consolidate the application of mechanotransduction in multiple fields.

## 10 Conclusions and Perspectives

The mechanotransduction and mechanobiology field has been shedding light on how mechanical stimuli are intricately and ceaselessly interlinked with life in different length scales. Specifically, mechanical forces can unfold proteins and regulate binding/unbinding dynamics, facilitating changes in genetics, epigenetics, subcellular architecture, and signaling transduction.^[^ ^20^ ^]^ These changes further induce short‐ and long‐term responses that optimize various cellular behaviors and remodel extracellular contexts. With further assistance from cell–cell, cell–matrix, and more complex communications, these responses are projected to impact embryogenesis, development, and physiology. This hierarchical regulation of sensing, transiting, amplifying, converting, and integrating diverse and dynamic mechanical stimuli, may be principal for the entire body to maintain functional and physiological homeostasis.

Despite these immense advances, insights into the intrinsic discrepancies and connections between mechanotransduction at different length‐scales remain rudimentary. A charming key to overcome this dilemma may be relevant to appreciating the origin and evolution of mechanotransduction. Conceivably, the long history of evolution is accompanied by the burgeoning capacity of living things to sense and communicate to a wide variety of stimuli from their surroundings. That is, multicellular organisms evolutionally develop complex hierarchical structures spanning from organ/tissue to subcellular scales, forming a prestressed network. Adherently, mechanical stimuli are considerably transmitted along this network to reach at the micro‐scale,^[^ ^29^, ^30^, ^441^ ^]^ varying with tempo‐spatial scales.^[^ ^441^ ^]^ In this regard, the complexity of mechanotransduction may evolve simultaneously with the organism's biology and mechanical stimuli. Intriguingly, recent evidence suggests that human cancer cells display certain atavistic features.^[^ ^442^, ^443^, ^444^, ^445^ ^]^ Therefore, it is of great interest to investigate the conservation and adaptation of mechanotransduction during evolution, which could definitely deepen our understanding on mechanobiology, as well as human physiology and pathology.

Mechanotransduction is reminiscent of numerous regulatory processes that function at different scales but integrate to accomplish one ultimate goal, in which it is vital to distinguish and interpret the instructive cues from mechanical noises. A better knowledge on this issue may rely on the advances of several aspects. First, it is important to dissect the dynamics of mechanoresponsive protein conformations and noncovalent bonds, as well as their subcellular and cellular compositions, given their significance on cellular sensitivity to mechanical stimuli.^[^ ^185^ ^]^ Second, exploration of the evolving mechanogenomic code, which ensures the homeostasis of cells and tissues in distinct physical contexts,^[^ ^17^, ^222^ ^]^ would be greatly valuable. Third, because energy is crucial for both physical information transformation and biological processes, it is interesting to elucidate the cross‐talk between mechanotransduction and metabolism.^[^ ^165^, ^169^ ^]^ Fourth, a plethora of regulatory loops conceivably functions across space‐spans from molecule to organ and time‐scales from milliseconds to months^[^ ^80^ ^]^ to maintain multifarious mechano‐biochemical equilibriums.^[^ ^241^, ^446^ ^]^ The new information is also critical for defining underlying mechanotransduction mechanisms. Last but not least, deregulated mechanotransduction are implicated in human diseases; however, current studies in this field mainly focus on the role of single or a few molecules and signaling pathways. An integrated investigation on hierarchical mechanotransduction across multiple scales, as well as their implications in classical and emerging therapeutic interventions,^[^ ^447^, ^448^ ^]^ are urgently needed.

Future investigations are still highly anticipated to reinforce present understandings and fill key gaps in theoretical and applicational regions. From the experimental perspective, mechanical contexts are viscous and crowded, and numerous biochemical responses proceed in ways of cascade amplification and induce a wide spectrum of micro and macroscopic outcomes. To this end, there is a need for establishing quantitative and robust tools capturing mechanobiology at different levels, such as enabling accurately and timely quantification and reconstitution of mechanical stimuli in vivo;^[^ ^449^ ^]^ synchronously reflecting multiple parameters in the mechanical conditions as in vivo; and employing the use of combined multi‐omics, including single‐cell spatial transcriptomics, and genome editing techniques to accurately dissect the spatiotemporal outputs of mechanotransduction. From the theoretical side, it is vital to enquire discrepancies in the mechanosensitivity of molecular, subcellular structures and cells in vitro and in vivo, genuine effects and in vivo mechanisms at divergent scales, and long‐term feedback or feedforward loops underlying multiscale mechanobiology. For the translational view, it is important to further improve the utilization of mechanical materials in 3D‐culture, organoid, and 3D/4D bioprinting, and also vastly expand novel mechanotransduction‐based therapeutics, ultimately harnessing disease and benefitting health.

## Conflict of Interest

The authors declare no conflict of interest.
